# The mRNA and miRNA transcriptomic landscape of Panax ginseng under the high ambient temperature

**DOI:** 10.1186/s12918-018-0548-z

**Published:** 2018-03-19

**Authors:** Inuk Jung, Hyejin Kang, Jang Uk Kim, Hyeonsook Chang, Sun Kim, Woosuk Jung

**Affiliations:** 1 Bioinformatics Institute, Seoul National University, Gwanak-Gu, Seoul, Republic of Korea; 2 Interdisciplinary Program in Bioinformatics, Seoul National University, Gwanak-Gu, Seoul, Republic of Korea; 3 National Institute of Horticultural and Herbal Science, Ginseng Research Division, RDA, Eumsung-gun, Chungbuk, Republic of Korea; 4 Department of Computer Science and Engineering, Seoul National University, Gwanak-Gu, Seoul, Republic of Korea; 5 Department of Applied Bioscience, Konkuk University, Gwangjin-Gu, Seoul, Republic of Korea

**Keywords:** Transcriptomic analysis, De novo assembly, Panax ginseng, Temperature

## Abstract

**Background:**

Ginseng is a popular traditional herbal medicine in north-eastern Asia. It has been used for human health for over thousands of years. With the rise in global temperature, the production of Korean ginseng (Panax ginseng C.A.Meyer) in Korea have migrated from mid to northern parts of the Korean peninsula to escape from the various higher temperature related stresses. Under the high ambient temperature, vegetative growth was accelerated, which resulted in early flowering. This precocious phase change led to yield loss. Despite of its importance as a traditional medicine, biological mechanisms of ginseng has not been well studied and even the genome sequence of ginseng is yet to be determined due to its complex genome structure. Thus, it is challenging to investigate the molecular biology mechanisms at the transcript level.

**Results:**

To investigate how ginseng responds to the high ambient temperature environment, we performed high throughput RNA sequencing and implemented a bioinformatics pipeline for the integrated analysis of small-RNA and mRNA-seq data without a reference genome. By performing reverse transcriptase (RT) PCR and sanger sequencing of transcripts that were assembled using our pipeline, we validated that their sequences were expressed in our samples. Furthermore, to investigate the interaction between genes and non-coding small RNAs and their regulation status under the high ambient temperature, we identified potential gene regulatory miRNAs. As a result, 100,672 contigs with significant expression level were identified and 6 known, 214 conserved and 60 potential novel miRNAs were predicted to be expressed under the high ambient temperature.

**Conclusion:**

Collectively, we have found that development, flowering and temperature responsive genes were induced under high ambient temperature, whereas photosynthesis related genes were repressed. Functional miRNAs were down-regulated under the high ambient temperature. Among them are miR156 and miR396 that target flowering (SPL6/9) and growth regulating genes (GRF) respectively.

**Electronic supplementary material:**

The online version of this article (10.1186/s12918-018-0548-z) contains supplementary material, which is available to authorized users.

## Background

Ginseng is a well-known traditional herbal medicine in north-eastern Asia and it has been used for human health for over thousands of years. The medicinal effects of ginseng have been reported as a preventative for various diseases such as cancer, cardiovascular disease, hepatotoxicity and aging related and central nervous system related neurodegenerative diseases [1]. Ginseng is a typical short day and annual plant that favors low light intensity and low temperature under 30 °C during the day period. Major locations of Korean ginseng (*Panax ginseng C.A.Meyer*) production in Korea have migrated from mid to northern parts of the Korean peninsula to escape from the various higher temperature related stresses. Such migration is causing shortage in farmland for cultivating ginseng, which is a common and current problem for worldwide ginseng growers.

High ambient temperature is usually defined as the condition of 3 to 6 °C higher than the normal temperatures. Under the high ambient temperature, vegetative growth is accelerated and results in early flowering [2]. In the growth chamber study, it has been reported that seed production was lower under higher temperature [3]. Similarly, the CO_2_ exchange rate usually increases in a higher temperature, resulting in a decrease of yield of ginseng root. There are a number of studies of the thermosensory pathway in Arabidopsis, however only few studies focused on food crops and perennial plants like ginseng [4]. Unlike annual plants, perennial plants may memorize some environmental stresses as a irreversible manner, which can transmit to the following years so that some tolerances against the stress that they have been exposed to could be acquired.

To investigate the biological mechanisms in ginseng that are affected under the high ambient temperature, we have cultivated *P. ginseng* plants in a temperature-gradient green house (TGG). Two samples, G3 and G5, were collected that were cultivated under normal temperature (24 °C) and high ambient temperature conditions (27 °C) respectively. From the collected samples, we performed small RNA and mRNA high throughput sequencing.

### Challenges in the analysis of transcript abundance without a reference Ginseng genome

Ginseng is a medicinal herb with high demands in east Asia. Existing studies so far mainly focused on the medicinal components of ginseng and their efficacy [5]. Ginseng is a tetraploid plant whose haploid genome is estimated to be 3.3 Gbp [6] and is known to have a genomic complexity involving a large number of major repetitive sequences, making it difficult to determine its genomic sequence. Only recently, genomic and transcriptomic studies using the high throughput sequencing technology were performed to reveal the large composition of major repeats in the genome [7], search for root specific miRNAs [8, 9] or understand the biological metabolisms in the Ginseng plant, such as the synthesis of ginsenosides [10] or the biological response to autotoxins [11]. However, to our knowledge, there is no study on how genes and miRNAs respond to the ambient hight temperature in *P. ginseng*.

The plant itself is largely understudied compared to its medical significance and demands worldwide. The lack of experimentally validated genes, proteins and non-coding RNAs of the *P. ginsen*g genome hinders the analysis of gene association, gene regulation or annotation of pathways or coding/non-coding transcripts. As of now, only 29 miRNAs specific to *P. ginseng* are deposited in miRBase [12]. Also, databases that archive curated or predicted genomic information of the ginseng plant are not present or not accessible.

The analysis of transcriptomic data mainly relies on aligning reads to a reference sequence, which is not available for the non-model plant species *P. ginseng* plant. Traditional sequencing was initially used to obtain root expressed sequence tags (ESTs) [13], which are very low in quantity. Studies based on high throughput sequencing data performed functional gene analysis based on contigs assembled from their sampled DNA or RNA libraries. In addition, the *P. ginseng* varieties differ between the contig sequences submitted to public databases. Thus, the quality of contigs assembled from a small number of samples and the sequence variance across sub-species makes it difficult to utilize them directly for sample or condition specific gene analysis. For genes, only partial gene sequences, such as ESTs [13] and assembled contigs [11] are available.

With the aforementioned challenges in preforming transcriptomic analysis of the *P. ginseng* plant, we have made several efforts to overcome them by the means of understanding the biological response of the plant under the influence of the high ambient temperature. The major tasks performed are categorized into three parts, which are described in more detail in the “Methods and Results” section in order as described below. First, we have collected a total of 65 paired end RNA-seq samples from the Sequence Read Archive (SRA) for the assembly of transcripts (Additional file 1). Among the assembled contigs, candidate protein coding transcripts were annotated based on detecting long open reading frames (ORF) and searching their closest orthologs in the NR [14] and UniRef90 [15] protein databases. Their expression levels were quantified by the high ambient temperature treated samples. Second, miRNAs were searched and quantified using our small RNA-seq data with the aid of miRBase and a de-novo miRNA detection tool. Third, associations between miRNAs and their candidate target transcripts were identified for functional analysis under the influence of the high ambient temperature. For validation purposes, several transcripts were selected, which sequences were validated by performing RT-PCR.

## Materials and experimental design

The goal of our study is to investigate biological mechanisms in ginseng that are affected under the high ambient temperature. In addition, we investigated the regulatory roles of miRNAs on mRNA transcripts under the high ambient temperature.

### Plant materials

*P. ginseng* (cv. Yeonpung) plants were cultured in a TGG located in Eumsung, Chungbuk of Republic of Korea. The micro climate inside of TGG was regulated for 24 h in the year round as described in [16] and [17]. The maximum temperature gradient in TGG was reached at 6 °C and the leaf samples for RNA extraction were obtained from two different locations of TGG, G3 and G5 which is near the front entrance and in the middle location of TGG, respectively. At the time of collecting the plant samples, the mean temperature of the front entrance of the TGG was 24 °C, which was the same as the outdoor temperature. The mean temperature in the middle location of the TGG was 27 °C. The temperature of the TGG was measured throughout the year at several locations within the TGG, which is shown in Additional file 2. The average temperature difference between G3 and G5 was 3 °C. Leaves of three years-old ginseng were obtained from three to five different plants in each G3 and G5 that were frozen immediately on site with liquid nitrogen on August 3 2016. Total RNAs of ginseng leaves were extracted using Qiagen RNA extraction kit (Qiagen USA). We used homogenizer to break the cell walls. Extraction of total RNA was repeated for three times where the samples for RNA sequencing were mixed in all three total RNAs repetitively. The sequencing were carried by the Macrogen company in Seoul, Korea.

## Methods

**Fig. 1:**
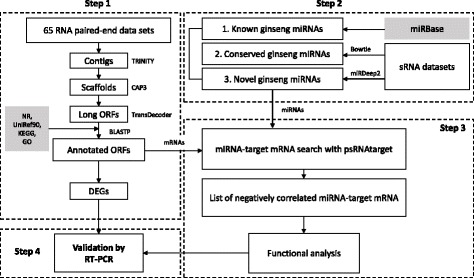
Analysis workflow. The analysis workflow consists of four steps: 1) the identification of putative coding genes, 2) miRNA detection, 3) predicting mRNA targets of miRNAs and 4) validating genes and miRNAs by RT-PCR. The gray boxes represent external databases used for the annotation of genes and miRNAs

To identify the high ambient temperature responsive mRNAs and regulatory miRNAs, our analysis was performed in four parts as shown in Fig. 1. First, contigs were assembled to search and annotate putative protein coding genes. Second, known, conserved and novel miRNAs with valid miRNA precursors were predicted from our assembled contig sequences. Third, negatively correlating expression levels between miRNAs and their target genes were searched. At last, selected genes that were predicted to have functional roles or are responsive to high ambient temperature were validated by reverse transcripted (RT) PCR.

In the first part of performing the transcriptomic analysis of the *P. ginseng* transcriptome data, we assembled and annotated high quality contigs utilizing 65 paired-end RNA-seq samples collected from SRA. For sample-specific contig sequences, we further corrected the assembled contigs using our RNA-seq samples. Here, gene expression was also quantified using our RNA-seq data using the assembled contigs. In the second part, miRNAs and their precursors were predicted using the assembled contigs. In the last part, we identified miRNA and their target genes that have negative correlating expression levels. The result of the workflow analysis is summarized in Table 1.

**Table 1:** As a summary of workflow analysis results, a total of 100,672 contigs were assembled of which 26,460 contigs were identified as protein coding genes

Gene set	number of genes
Contigs	100,672
Protein coding contigs	26,460
DEGs	1256
DEGs with enriched GO terms	752
DEGs with enriched pathways	241

From the protein coding genes, 1256 genes were detected as DEGs. From the DEGs, 73 enriched GO terms were present involving 752 genes. Also, from the DEGs, 22 enriched pathways were present involving 241 genes

## Results

### Part1: Contig analysis for sample specific gene annotation

For species without a fully annotated reference genome like *P. ginseng*, *de novo* assembly must be performed for identifying genes and for further downstream gene expression analysis. Previous studies performed EST sequencing, whole genome sequencing and transcriptome sequencing. None of them are enough to complete the complex Ginseng genome. Furthermore, the subspecies also differ between the studies. Hence the studies performed sample specific contig assembly utilizing only a small number of samples (i.e., a maximum of up to 12 RNA-seq samples) [11]. Since the sequence variation between the existing contigs and EST sequences are large, the RNA-seq alignment are poor, which may not be used for designing primer sequences during sequence validation. In this study, we utilized as many available *P. ginseng* high throughput samples as possible to assemble high quality contigs, which are then subsequently corrected by building consensus sequences using sample specific RNA-seq reads. In this manner, we were able to mask out sequence variations caused by sub-species differences and perform an effective downstream analysis to reveal biological mechanisms under the high ambient temperature.

The assembly process generated a total of 100,672 contigs. From the contigs, a total of 34,497 protein coding genes were annotated through the sequence similarity search of identified open reading frames (ORF) against the non-redundant protein NR and UniRef90 databases. Differentially expressed gene (DEG) analysis and the gene set enrichment analysis were performed to identify enriched GO (Gene Ontology) terms and pathways.

#### *De novo* assembly

From the SRA database, 65 paired end RNA-seq data sampled from *P. ginseng root*, stem and leaf tissues were collected. Reads from the collected data were pooled together and assembled into the new reference transcriptome sequences. The transcriptome assembly was performed using Trinity (version 2.1.1) [18]. To be able to process the large RNA-seq data sets, we used the Trinity *in silico* normalization. All parameters were set as default. As a result 62.9 million base pairs and 744,507 contigs were assembled. We further performed scaffolding using CAP3 [19] to acquire a total of 613,448 scaffolded contigs. To compensate for sequence variations caused by sub-species difference, our transcriptome RNA-seq reads were used to build sample specific consensus sequences. As a result, the N50 was 845 bp and the average contig length was 625 bp. The GC content was 36.01%.

#### Measuring contig expression levels

To measure the expression levels of contigs, we aligned the paired RNA-seq reads from G3 and G5 samples using Bowtie2 [20] to the contigs. For the alignment options, we discarded unpaired and discordant paired reads. Subsequently, we computed FPKM (Fragments per kilobase of exon per million fragments mapped) of each contig for quantifying their expression levels. Contigs with expression level below 1 FPKM were not considered for further analysis.

#### Annotation of expressed transcripts in contigs

For the functional annotation of contigs, we performed similarity search of the contigs against the NR and UniRef90 non-redundant protein databases. We first extracted putative coding regions from the assembled contig sequences using TransDecoder, which is a Trinity software package. A total of 34,497 protein sequences with a minimum length of 100 amino acids were collected. These peptide sequences were searched using BLASTP with an E-value < 10^−6^ against the protein databases. Using the UniProtKB [21] and GO database [22], we further annotated genes with their associated KEGG IDs and GO terms. A total of 26,460 genes were annotated. The list of annotated genes including their searched orthologs in other plant species are provided in the Additional file 3.

#### Identifying differentially expressed genes

**Fig. 2:**
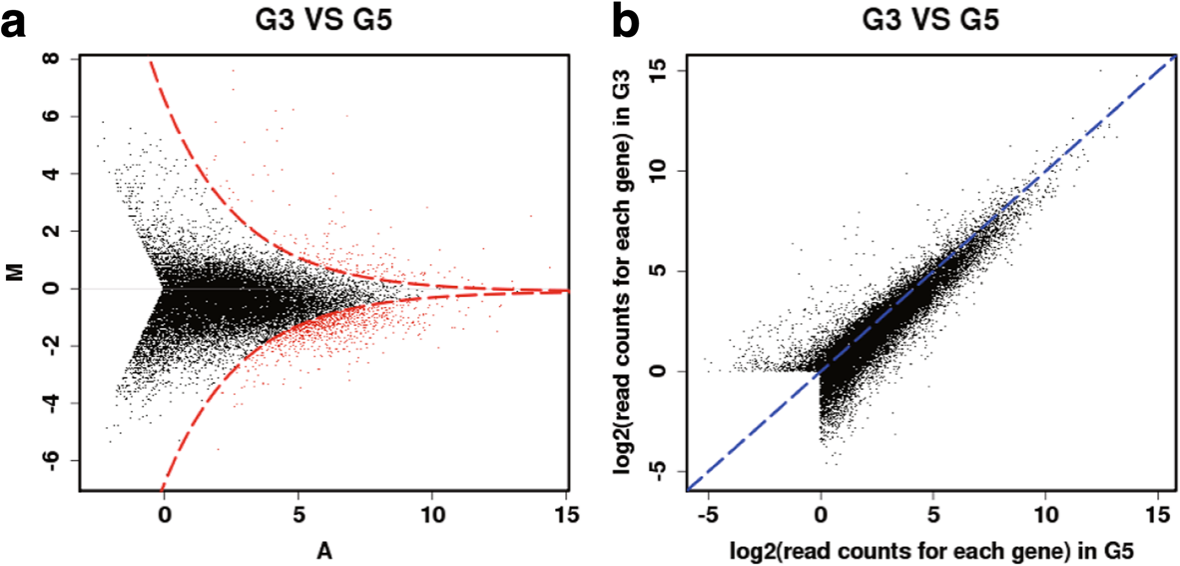
DEG analysis plot results. **a** MA-plot of genes in G3 and G5. The DEGs are shown in red dots. **b** Expression level of each gene in G3 and G5 samples. It shows that a larger portion in G5 have higher expression levels compared to G3, which is also observed in the portion of up-regulated DEGs

To identify genes that are differentially expressed under the high temperature, we used DEGseq [23], which detects DEGs based on the MA-plot method with a random sampling model. A stringent cut-off value of *P*<0.001 was for DEG detection. Multiple testing correction has been applied during the DEG detection process (i.e., q-values are included in the Additional file 4, which uses the Benjamini-Hochberg procedure). A total of 1256 DEGs were identified where 1028 and 228 genes were significantly up and down regulated in G5 compared to G3, respectively. The MA-plot is shown in Fig. 2. As shown, most of the DEGs are induced under the high ambient temperature.

We categorized differentially expressed genes into three groups: Photosynthesis related genes, temperature responsive genes and flowering/developmental related genes. The photosynthesis related genes were down regulated under the high ambient temperature (Table 2). On the other hand, temperature responsive and flowering and growth related genes were induced under the high ambient temperature (Table 3). The expression level and significance of differentially expressed genes are provided in the Additional file 4.

**Table 2:** List of photosynthesis related genes that are down regulated under high ambient temperature

Database	Contig ID	G3	G5	FC	DEG	Protein description	Protein symbol
UniRef90	TRINITY_DN310811_c0_g1_i1	3.985	3.581	0.27	O	Photosystem II CP43 reaction center protein	psbC CP43
UniRef90	TRINITY_DN310811_c0_g2_i1	4069.172	3630.229	0.281	O	Photosystem II CP43 reaction center protein	psbC CP43
UniRef90	Contig5643	191.474	162.955	0.365	O	Photosystem II CP47 reaction center protein	psbB
UniRef90	Contig14080	2556.7	1979.351	0.541		Photosystem II CP43 reaction center protein	psbC CP43
UniRef90	Contig14080	9.28	6.867	0.541		Photosystem II D2 protein	psbD
UniRef90	TRINITY_DN187814_c0_g1_i1	1027.871	708.731	0.563		Photosystem II CP47 reaction center protein	psbB
UniRef90	TRINITY_DN211582_c2_g7_i1	749.004	512.517	0.624		Root phototropism protein, putative	RCOM_1092950
UniRef90	TRINITY_DN214723_c1_g1_i1	961.187	652.51	0.676		Photosystem II reaction center Psb28 protein	
UniRef90	Contig17873	98.53	66.629	0.679		Photosystem II 5 kD family protein	POPTR_0009s05720g
UniRef90	TRINITY_DN178366_c1_g1_i1	4.157	2.593	0.684		Putative photosystem I reaction center subunit X	
UniRef90	TRINITY_DN216985_c9_g2_i1	3.517	1.98	0.69		Photosystem II 10 kDa polypeptide family protein	POPTR_0001s42970g
UniRef90	TRINITY_DN71881_c0_g1_i1	34.577	18.701	0.74		Photosystem I P700 chlorophyll a apoprotein A2	RCOM_2119600
UniRef90	TRINITY_DN179684_c0_g1_i1	34.577	18.701	0.774		Photosystem I reaction center subunit II, chloroplast	RCOM_0677760
UniRef90	TRINITY_DN214723_c1_g2_i1	36.482	13.331	0.851		Photosystem II reaction center Psb28 protein	
UniRef90	TRINITY_DN198228_c4_g1_i1	9.8	2.752	0.892		Photosystem I reaction center subunit VI	RCOM_1493780
UniRef90	Contig14782	20.642	5.583	0.899		DNA photolyase	At4g25290

FC is the fold-change of contigs (G5/G3)

**Table 3:** List of temperature responsive genes that are induced under high ambient temperature

Database	Contig ID	G3	G5	FC	DEG	Protein description	Protein symbol
UniRef90	Contig12068	47.314	115.989	2.451	O	Low temperature and salt responsive protein family	RCI2B
UniRef90	TRINITY_DN194823_c4_g2_i1	1.183	2.434	2.057	O	low-temperature-responsive protein 78	LTI78
UniRef90	TRINITY_DN215430_c2_g10_i1	52.944	71.743	1.355		temperature-induced lipocalin	ATTIL, TIL
NR	Contig13986	13.059	21.587	1.653		temperature sensing protein-related	EDA35
UniRef90	TRINITY_DN212719_c3_g5_i1	22.746	27.332	1.202		staurosporin and temperature sensitive 3-like b	STT3B
NR	Contig13986	13.059	21.587	1.653		temperature sensing protein-related	EDA35
UniRef90	Contig18568	59.544	167.28	2.809	O	soybean gene regulated by cold-2	SRC2
UniRef90	TRINITY_DN204672_c3_g4_i1	81.603	185.102	2.268	O	cold shock domain protein 1	CSDP1
UniRef90	TRINITY_DN143765_c1_g1_i1	3344.374	6457.804	1.931		cold, circadian rhythm, and RNA binding 1	CCR1
UniRef90	TRINITY_DN143765_c1_g1_i1	3344.374	6457.804	1.931		cold, circadian rhythm, and rna binding 2	CCR2
NR	Contig7422	32.93	58.654	1.781		cold shock domain protein 3	CSP3
UniRef90	TRINITY_DN180886_c3_g1_i1	53.957	87.33	1.619		cold regulated 413 plasma membrane 1	COR413-PM1
UniRef90	Contig29838	115.723	141.657	1.224		COLD REGULATED 314 INNER MEMBRANE 1	COR414-TM1
UniRef90	Contig29838	115.723	141.657	1.224		cold regulated 413 plasma membrane 1	COR413-PM1
UniRef90	Contig6806	655.021	798.658	1.219		cold-regulated 47	COR47
NR	Contig30457	62.891	52.302	0.832		cold regulated gene 27	
NR	Contig30457	62.891	52.302	0.832		cold regulated gene 27	
UniRef90	Contig7573	71.504	241.895	3.383	O	Heat shock cognate 70 kDa protein 2	HSC-2
UniRef90	TRINITY_DN206978_c4_g7_i1	2.102	6.909	3.287	O	Heat shock protein SSB1	CTRG_03108
UniRef90	TRINITY_DN211503_c10_g1_i1	2.945	9.343	3.172	O	Heat shock protein 70 cognate	POPTR_0001s03410g
UniRef90	Contig10827	9.612	27.397	2.85	O	Heat shock protein 70	Hsp70
UniRef90	Contig12226	5.551	15.352	2.766	O	Heat shock protein 70	Hsp70
UniRef90	Contig27208	2.198	6.016	2.737	O	Heat shock protein 70 cognate	POPTR_0001s03410g
UniRef90	Contig491	21.018	56.678	2.697	O	Heat shock protein 70	Hsp70
UniRef90	Contig41549	13.056	32.822	2.514	O	Heat shock protein 90-2	HSP90-2
UniRef90	Contig7574	123.881	295.909	2.389	O	Heat shock protein 70 (Fragment)	Hsp70
UniRef90	Contig13144	2.657	6.189	2.329	O	Heat shock protein 90	NtHsp90er-2
UniRef90	TRINITY_DN211503_c13_g8_i1	6.785	15.285	2.253	O	Heat shock cognate protein	SCHSP70
UniRef90	TRINITY_DN211503_c13_g6_i1	5.548	12.377	2.231	O	Heat shock cognate 70 kDa protein 2	HSC-2
UniRef90	Contig7001	5.285	11.628	2.2	O	Heat shock protein 60	
UniRef90	TRINITY_DN216671_c13_g21_i1	8.133	17.801	2.189	O	Heat shock protein 70-like protein	Hsp70
UniRef90	TRINITY_DN177960_c1_g1_i1	9.841	21.242	2.159	O	Heat Stress Transcription Factor family protein	POPTR_0016s05680g
UniRef90	Contig13383	63.889	137.162	2.147	O	Heat shock factor	NtHSF1
UniRef90	Contig42189	2.738	5.664	2.069	O	Heat shock protein 70	POPTR_0008s15140g
UniRef90	Contig19499	47.77	96.941	2.029	O	Heat shock protein DnaJ	POPTR_0017s08520g
UniRef90	TRINITY_DN207002_c3_g1_i1	28.896	58.041	2.009	O	Heat shock protein 70 cognate	POPTR_0001s03410g
UniRef90	Contig5684	9.573	19.091	1.994		Heat shock protein 70-like protein	Hsp70
UniRef90	TRINITY_DN203097_c2_g2_i1	10.877	19.739	1.815		Heat shock protein, putative	RCOM_1582180
UniRef90	TRINITY_DN22543_c1_g1_i1	2.424	4.359	1.798		Heat shock protein 90	NtHsp90er-2
UniRef90	TRINITY_DN208763_c5_g5_i1	1.235	2.108	1.707		Heat shock protein 70	RCOM_0911270
UniRef90	TRINITY_DN163361_c0_g2_i1	1.609	2.741	1.704		Heat shock 70 kDa protein, mitochondrial	HSP68
UniRef90	TRINITY_DN213169_c3_g3_i1	3.711	6.094	1.642		Heat shock cognate 70 kDa protein	LOC_Os11g47760
UniRef90	TRINITY_DN190866_c6_g4_i1	4.813	7.637	1.587		Heat shock protein binding protein, putative	RCOM_1732070
UniRef90	TRINITY_DN163361_c0_g1_i1	1.814	2.832	1.561		Heat shock 70 kDa protein, mitochondrial	Hsp68
UniRef90	TRINITY_DN195294_c1_g1_i1	1.402	2.148	1.532		Heat shock 70kD protein (Fragment)	Hsp70
UniRef90	TRINITY_DN200501_c3_g4_i1	1.395	2.016	1.445		Heat shock protein 90 (Fragment)	M569_09035
UniRef90	Contig30036	2.547	3.653	1.434		Heat shock protein 90 (Fragment)	Hsp90
UniRef90	Contig4365	18.055	24.216	1.341		Heat shock cognate protein	SCHSP70
UniRef90	TRINITY_DN216543_c12_g10_i1	11.667	15.016	1.287		Heat shock protein 60 (Fragment)	hsp60
UniRef90	Contig20702	16.978	21.647	1.275		Heat shock cognate 70 kDa protein	LOC_Os11g47760
UniRef90	Contig34911	9.443	12.021	1.273		Heat shock protein 70	Hsp70
UniRef90	Contig28357	3.003	3.812	1.269		Heat shock 70 kDa protein 10	HSP70-10
UniRef90	TRINITY_DN213351_c2_g3_i1	3.194	3.823	1.197		Heat shock factor protein 5	POPTR_0010s11490g
UniRef90	Contig42828	2.283	2.632	1.153		Heat shock protein binding protein, putative	RCOM_0564980
UniRef90	TRINITY_DN195327_c8_g5_i1	2.726	2.974	1.091		Heat shock 70 kDa protein 7	HSP70-7
UniRef90	TRINITY_DN208988_c4_g4_i1	12.901	13.632	1.057		Heat shock protein 70	POPTR_0001s29210g
UniRef90	Contig8566	10.542	10.921	1.036		Heat shock protein	ophh-56
UniRef90	TRINITY_DN174225_c0_g1_i1	1.589	29.996	18.877	O	Putative uncharacterized protein	FPF1
UniRef90	Contig1416	8.015	21.611	2.696	O	Uncharacterized protein	
UniRef90	TRINITY_DN194926_c35_g60_i1	45.891	111.128	2.422	O	Putative uncharacterized protein	LHY
UniRef90	Contig1941	12.978	28.424	2.19	O	Beta-amylase (EC 3.2.1.2)	
UniRef90	TRINITY_DN211505_c5_g1_i1	7.987	15.501	1.941		WRKY5	FT
UniRef90	TRINITY_DN212536_c5_g1_i1	9.007	12.199	1.354		Gigantea-like	GI
UniRef90	TRINITY_DN210901_c7_g2_i1	32.791	42.343	1.291		Putative uncharacterized protein	TOC1
UniRef90	TRINITY_DN212865_c9_g6_i1	1.239	2.948	2.379	O	Phototropin-2	PHOT2
UniRef90	TRINITY_DN216565_c8_g14_i1	1.612	2.838	1.761		Phototropin-1	M569_09651
UniRef90	Contig7059	1.487	2.447	1.646		Phototropin-2	PHOT2
UniRef90	TRINITY_DN143824_c1_g1_i1	370.609	458.745	1.238		Chloroplast photosynthetic water oxidation complex 33kDa subunit	
UniRef90	TRINITY_DN207410_c1_g1_i1	1238.763	1242.461	1.003		Photosystem I reaction center subunit IV A	MTR_7g099390

FC is the fold-change of contigs (G5/G3)

#### Gene set enrichment functional analysis

The functional analysis of DEGs were performed by the GO and the pathway enrichment analysis. For both enrichment analysis, we performed Fisher’s exact test with *P*<0.05. For the GO enrichment test, we used genes with annotated GO terms as the background set. A total of 45,075 unique GO terms were present in the background set. The list of DEGs with annotated GO terms were used as the query set, where 3,966 unique GO terms were present. 73 GO terms showed a significant enrichment. The two most highly enriched GO terms were ‘ATP binding’ related GO terms (i.e., ‘ATP binding’ and ‘Chloroplast thylakoid membrane’). Light harvesting related GO terms, such as ‘Protein-chromophore linkage’, ‘Photosystem I/II’, ‘Photosynthesis’, ‘Light harvesting in photosystem I’ and ‘Pigment binding’ were next highly enriched. The list of enriched GO terms are provided in Additional file 5(a).

For the pathway enrichment test, we used genes with annotated KEGG pathway IDs (i.e., KO IDs) as a background set. A total of 2,415 unique KO IDs were present in the background set. The list of DEGs with annotated KO IDs were used as the query set, where 260 unique KO IDs were present. 22 pathways showed a significant enrichment of DEGs. There were a significant number of DEGs in two photosynthesis pathways (i.e., ‘Photosynthesis’ and ‘Photosynthesis - antenna proteins’), and the ‘Plant hormone signal transduction’ pathway. A majority of genes, including DEGs that mapped to photosynthesis pathways, were significantly down regulated. Within the ‘Plant hormone signal transduction’ pathway, the genes along its sub-pathways related to Auxin (i.e., AUX1, TIR1, ARF etc.), Giberrellin (i.e., GID1, GID2, DELLA, TF) and Abscics acid (i.e. PYR/PYL, PP2C and SnRK2), were significantly up regulated. The three sub-pathways are shown to induce cell enlargement, plant growth, stem growth or germination and stomatal closure and seed dormancy respectively. The list of enriched pathways is shown in the Additional file 5(b).

#### Validation of contigs using RT-PCR

In order to validate and check the quality of assembled contigs, we selected six contig sequences genes, that have significant homology with Arabidopsis genes (i.e., putative ginseng 2-oxoglutarate, PIF4, PCK1, R3H, FPF1 and ERF72), and synthesized primers to amplify a certain part of the selected genes. The pair primer sequences for a certain part of the six genes are as follows: ‘AACGCCGGTCCT’ and ‘ATGCAATTCTAGCCAG’ for 2-oxogltarae, ‘AGCCACAGATCCCC’ and ‘AAGGTTCTGAGCATG’ for PIF4, ‘CAGTTCCCGTGT’ and ‘GCTGGTCCTATATTCT’ for PCK1, ‘CAAAACCCTCGTCA’ and ‘TGACGCCTTTTAATATG’ for R3H, ‘CGGAAAATGCTAGTCCA’ and ‘GTCGTTAAGAATCGTAA’ for flowering promoting factor 1, ‘CTCGACGGATCCAC’ and ‘GGGTTTGGCTGACTC’ for ERF72.

The first cDNA strand was synthesized by RT-PCR which was carried out with RT-PCR kit of Invitrogen using both gene specific primers. In addition, we used a gene specific primer for upper primer and oligo (dT) for down primer to amplify 3 ^′^-end regions and to compare with contigs we constructed as described.

Among the six sequences, the contig sequence aligning to R3H showed a clear band during the validation. As a result, two sequences with length of 665 and 657 nt were detected (Additional file 6 - Sequence 1 and Sequence 2). The sequences were BLASTN searched in our contig set. Each sequence aligned on two different contigs with significant E-values, which are TRINITY_DN215770_c7_g28_i1 (E-value =0.0) and Contig8260 (E-value =4*e*^−71^). The two contigs commonly embed the R3H domain, which is reported to be involved in polynucleotide-binding, including DNA, RNA and single-stranded DNA. This is due to its association with the AAA domain or with various DNA/RNA binding domains, such as DSRM, KH, G-patch, PHD, DEAD box or RRM. Especially, the R3H domain is reported to stabilize poly(A)-specific ribonuclease by stabilizing the RRM domain [24]. The BLASTX search result on the Arabidopsis TAIR 10 database showed similar results, where both synthesized sequences showed a significant hit to single-stranded nucleic acid binding R3H proteins, such as AT3G56680. The contig TRINITY_DN215770_c7_g28_i1 well aligns to the Phytoclock Like 1 (PCL1) protein sequence, which encodes a MYB family transcription factor with a single MYB DNA-binding domain (type SHAQKYF) that is unique to plants and is essential for circadian rhythms, specifically for transcriptional regulation within the circadian clock. It is also known as the symbol LUX, which is required for normal rhythmic expression of multiple clock outputs in both constant light and darkness. It is co-regulated with TOC1 and seems to be repressed by CCA1 and LHY by direct binding of these proteins to the evening element in the LUX promoter [25]. In A. thaliana, PCL1 is also known to encode a novel GARP protein that is essential for the circadian clock [26]. While not discussed with further detail, three other genes were successfully synthesized, which were the contigs that well aligned to MET1, DDM1 and Chlorophyll A/B binding protein encoding gene sequences (Additional file 6 - Sequence 3, 4 and Sequence 5).

### Part2: Sample specific small non-coding RNA analysis

To investigate how small RNAs interact with genes and regulate gene expression level under the high ambient temperature, we searched and predicted miRNAs from the small RNA-seq data. Among the annotated ginseng miRNAs in miRBase, 6 miRNAs were searched with significant expression levels. A total of 214 miRNAs conserved in other species were detected. In addition, 60 novel miRNAs with associated template precursors were predicted from our assembled contigs. Interestingly, functional miRNAs showed down regulation under the high ambient temperature. Consistently, their target genes were up regulated.

#### Landscape of small RNA transcripts in *P. ginseng*

**Fig. 3:**
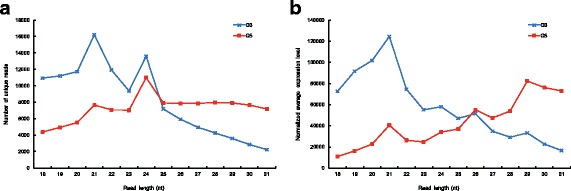
Features of the small RNA population by transcript length. **a** The number of unique transcripts were the greatest for 21 nt transcripts. G3 and G5 both showed a peak for 24 nt transcripts. The population of unique reads increased with the length of transcripts in G5, whereas it declined in G3 for transcripts longer than 25 nt. **b** The expression level of small RNA transcripts similarly showed a spike at 21 nt. Also, the expression level increased with the length of small RNA transcripts specific to G5

We found that the landscape of small RNA transcripts significantly differed in G3 and G5 as shown in Fig. 3. In G3, small RNAs of 21 nt were most abundant while it was not the case in G5. Interestingly, longer small RNAs were expressed more in G5, which is a quite strong trajectory. The expression level of miRNAs was quantified by the miRNA read counts normalized by each sample size. As of now, our scientific community has limited knowledge on the biological mechanisms of *P. ginseng* and the landscape of small RNA transcripts needs in-depth investigation in the future. We discussed the regulatory roles of miRNAs in the “Discussion” section.

#### Predicting miRNAs

**Fig. 4:**
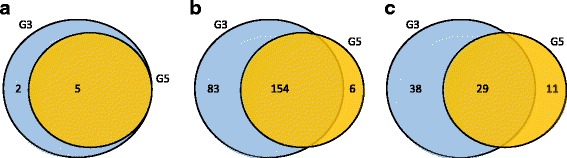
The number of detected miRNAs in G3 and G5 samples. **a** The number of known miRNA detected in G3 and G5 samples **b** The number of conserved miRNAs detected in G3 and G5 samples. **c** The number of novel miRNAs detected in G3 and G5 samples. The known miRNAs are the *P. ginseng* annotated miRNAs archived in miRBase. The conserved miRNAs are miRNAs that have a perfect sequence match in other plant species. At last, the novel miRNAs are miRNA sequences that are paired with a valid miRNA precursor detected by miRDeep2 from the assembled contig set

A total of 329 miRNAs were detected from the small RNA-seq samples. Eight known, 243 conserved and 78 novel miRNA transcripts were annotated as shown in Fig. 4.

#### Identification of known miRNAs

**Fig. 5:**
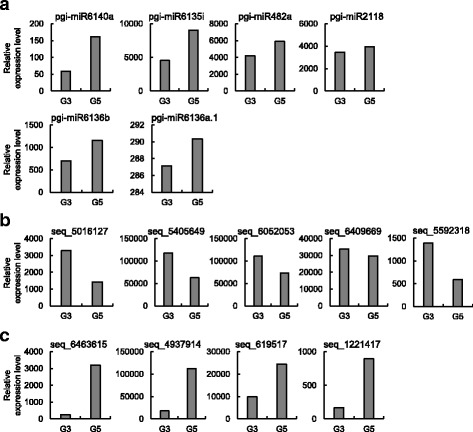
Expression levels of selected miRNAs. Here, sample size normalized expression level of selected miRNAs in G3 and G5 are shown. **a** The known *P. ginseng* miRNAs are induced under the high ambient temperature. **b** Novel and conserved miRNAs that are repressed under the high ambient temperature

Currently, 29 miRNAs, with precursor sequences, of the *P. ginseng* plant are annotated in miRBase [12], which were archived by the previous study [27]. Among them, five miRNAs were commonly expressed in G3 and G5 samples. All five miRNAs were up-regulated in G5. pgi-miR6140a was induced by more than 2-fold as shown in Fig. 5a. Furthermore, we tested if miRDeep2 [28] reported the known miRNAs, and their precursor sequences by providing the annotation information of the known miRNAs from miRBase. Among the five miRNAs, four miRNAs with associated precursors were detected.

#### Identification of novel miRNAs

Using miRDeep2, we searched for novel miRNA precursors and associated mature miRNA sequences from the assembled contigs. First, miRDeep2 aligned the small RNA-seq reads to the contigs without allowing any mismatches. Then it predicted RNA secondary structure from the RNAfold result, which predicts and scores RNA secondary structures of potential miRNA precursors. Potential miRNA precursors were determined by the following three criteria: (1) contigs that fold into an unbifurcated hairpin, (2) contigs that consist of mature (or core miRNA region), loop and star regions, and (3) contigs where 60% of reads map to the mature sequence region. As a result, we identified 101 and 82 novel miRNA precursors in G3 and G5, respectively. In total, 139 novel miRNA precursors were identified where 44 were commonly present in G3 and G5.

#### Identification of conserved miRNAs

**Fig. 6:**
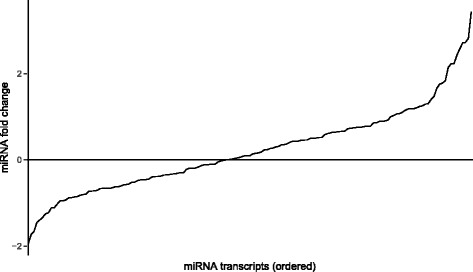
Expression fold change of identified miRNAs. The expression level fold change of the identified 156 miRNAs are shown in *log*_2_ scale. The miRNAs are sorted in ascending order of the *log*_2_ fold change

To identify conserved miRNAs in our G3 and G5 samples, we aligned the small RNA reads against the miRBase miRNA precursors allowing up to 2 mismatches. The short reads were aligned to the hairpins in miRBase that are annotated from more than 206 species. Species with less than 28 annotated miRNA hairpins were filtered out from the alignment, which accounts for 0.1% of the total annotated hairpins in miRBase. The organisms were sorted by the number of aligned reads to the associated organism’s hairpin sequence. The top 16 organisms were all plant species. Among them, the *P. ginseng* had the highest hits, which was very surprising in respect to the small number of annotated miRNA hairpins (i.e., 32 hairpin sequences). The following plants followed in descending order: L. usitatissimum, M. esculenta, T. cacao, R. communis. Similar to the known and novel miRNAs, the conserved miRNAs also showed a strong down-regulation pattern in G5. These conserved miRNAs in multiple organisms may share common functions, which can be valuable knowledge in *P. ginseng*. The fold-changes of miRNAs are shown in Fig. 6.

### Part3: mRNA-miRNA integrated analysis

To search for gene regulatory miRNAs, we identified target contigs of predicted miRNAs using psRNATarget [29]. psRNATarget is a small RNA target analysis tools for plant species. For the miRNA sequence input, we provided the known, conserved and novel miRNAs that are present in our small RNA-seq data. For the miRNA target candidates, we provided the protein coding contig sequences. Target contigs were selected as candidates when contigs with target sites have significant alignment with a miRNA and have strong target-site accessibility based on unpaired energy between nucleotides of the target site and the miRNA. Finally, we selected miRNA-target gene pairs only when the expression level were negatively correlated. As a result, a total of 592 miRNA and their target contig pairs with negative correlation were identified. Among them, a majority of 583 miRNAs were down regulated while their target genes were up regulated. Only 12 pairs were present where miRNAs showed mRNA suppression.

**Fig. 7:**
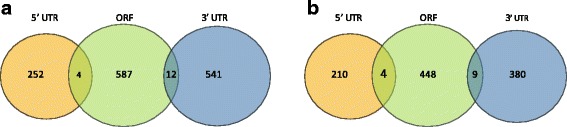
miRNA target sites. The number of miRNAs targeting each feature are shown in **a** G3 and **b** G5

We further investigated the miRNA target regions in the contigs. The target site statistics are grouped by genomic regions such as 3 ^′^ UTR, ORF or 5 ^′^ UTR region. From our assembled contigs, non-ORF regions that are located to the left of the starting ORF are annotated as 5 ^′^ UTR regions. Similarly, the non-ORF regions located at the right of the last ORF region are annotated as 3 ^′^ UTR regions. In summary, miRNA target sites seem to be distributed somewhat evenly across different regions within the gene body as shown in Fig. 7.

## Discussion

From hight-throughput sequencing data collected from the *P. ginseng* plant, we assembled transcript contigs and identified three types of gene sets that showed response to the high ambient temperature: 1) Heat stress associated genes, 2) Photosynthesis and respiration associated genes and 3) Flowering associated genes. Genes related to development and flowering were significantly induced under the high ambient temperature. Among all genes, the Flowering promoting factor 1 (FPF1) was the most up-regulated gene with 29-fold change in G5 compared to G3. The Phytochrome interacting factor 4 (PIF4) was also present in our contigs list, which was also up-regulated by 2.2-fold in G5. Interestingly, the target genes of PIF4, FT [30], was also up-regulated by 1.94-fold in G5. Brassinazole-resistant1 (BZR1) is known to be a co-interacting gene that interacts with PIF4 [31], which was also up-regulated by 2.7-fold.

A strong gene expression trend was observed between temperature (i.e., heat and cold) responsive and photosynthesis related genes. 14 low and cold temperature responsive genes were expressed, of which five were detected as up-regulated DEGs. The remaining genes were not DEGs but were all induced by some level. For example, the Low temperature induced 78 (LTI78) protein encoding gene was induced by more than 4-fold in G5. LTI78 is reported to accumulate in all genotypes in response to low temperature and drought and it is always present when plants were freezing tolerant in the A. thaliana organism [32]. Cold and circadian rhythm related genes, such as CDSP1, CCR1 and CCR2, are induced by 4 to 8-folds in G5. A total of 46 heat related genes were expressed, of which 40 were induced. Among the induced heat associated genes, 19 were DEGs. Only two down-regulated DEGs were detected. Most of the genes were Heat shock proteins where Hsp70 and Hsp90 were the most abundant ones. On the contrary, a majority of photosynthesis related genes were repressed. Among the 21 photosynthesis related genes, 16 were down-regulated, of which 3 were DEGs. Only one up-regulated DEG was detected. Phototropin, Photosystem I and II were the most abundantly found genes in the photosynthesis related gene set. Collectively, most of the temperature responsive genes were induced under the high ambient temperature, whereas photosynthesis related genes were down-regulated. The relation between temperature response and photosynthesis activity related genes showed opposite expression trends.

miRNAs that are associated to stress response and floral development are observed to be down-regulated under the high ambient temperature. Three stress responsive miRNAs conserved in other plant organisms (i.e., miR394, miR399a and miR8175) were expressed in large quantity that were all down-regulated in G5. They are known to be associated with salt and drought response in plants. The development associated miRNAs were also down-regulated. The miR156 is reported to be induced under stressful conditions in plants that adopted stress tolerant phenotypes [33]. SPL6 and SPL9 are among the targets of miR156, and when suppressed by miR156, the flowering time is delayed until a suitable conditions is met [34]. We observed that miR156 was repressed in G5 by 3-fold, whereas its target genes, SPL6 and SPL9, were induced correspondingly, which may imply that the flowering mechanism has been promoted under high ambient temperature. The development associated miRNAs miR159, miR319, miR390a and miR396. miR396 were down-regulated. They are known to antagonize the expression of the growth regulating factor (GRF) gene, which is a transcription factor. All of the GRF genes were induced, including the GRF2 to GRF12 genes. Collectively, the growth of the plant may have been accelerated by several biological mechanisms that includes the repression of miRNAs that target growth promoting genes [35].

In previous studies, the expression levels of small interfering RNAs (siRNAs) are shown to be dramatically reduced with the rising temperature [36]. However, the direction of expression pattern of miRNAs in response to stress are more dynamic. In the study [37], the miR169 was shown to be down-regulated in response to drought in A. thaliana, but up-regulated in rice.

## Conclusions

This study provides the analysis of mRNA and miRNA transcriptomic landscape of the Korean ginseng when treated with high ambient temperature. Especially, high quality contigs were assembled using a large number of publicly available data sets to correctly quantify their expression levels. Furthermore, miRNAs were predicted to observe their role under high ambient temperature, which is the first study of mRNA-miRNA integrated analysis under high ambient temperature in *P. ginseng* to our knowledge. This study has found that temperature responsive and flowering/growth related genes were induced under high ambient temperature, whereas photosynthesis related genes were repressed. Coherently, miRNAs that are predicted to target GRF (i.e., Growth regulating factor) genes were suppressed under high ambient temperature. The results and genetic resource of this study will provide valuable information in profiling temperature responsive genes in *P. ginseng*.

## Additional files

Additional file 1 The list of SRA samples used for contig assembly. (XLS 18 kb)

Additional file 2 Changes in daily average air temperature in the Temperature Gradient Greenhouse. (DOC 106 kb)

Additional file 3 Annotation of assembled contigs. The list of 26,461 contigs with significant E-value using BlastX are shown. The NR and UniRef90 protein sequence databases are used for the contig search. (XLS 3270 kb)

Additional file 4 Gene expression of assembled contigs. The expression levels and expression profiles of 26,461 contigs are shown. Also, the significance level of differential expression between G3 and G5 samples are provided. (XLS 2250 kb)

Additional file 5 GO and pathway enrichment analysis of contigs. The contigs were tested for any GO and pathway enrichment. The list of significantly enriched GOs are shown in (a). The list of significantly enriched pathways are shown in (b). (XLS 16 kb)

Additional file 6 Results related to sanger sequencing of asssembled contigs. The primer sequences, sanger sequence of selected contigs and BLAST results on NR and UniRef90 databases are shown. (DOC 733 kb)
