# Supplementary material for: The mRNA and miRNA transcriptomic landscape of Panax ginseng under the high ambient temperature
Source: BMC Syst Biol. 2018 Mar 19;12(Suppl 2):27. doi: 10.1186/s12918-018-0548-z (PMC5861484; doi:10.1186/s12918-018-0548-z)
Supplement: Supplementary file 2 — Changes in daily average air temperature in the Temperature Gradient Greenhouse. (DOC 106 kb) [file 12918_2018_548_MOESM2_ESM.docx]

**Additional file 2 – Figure S1**


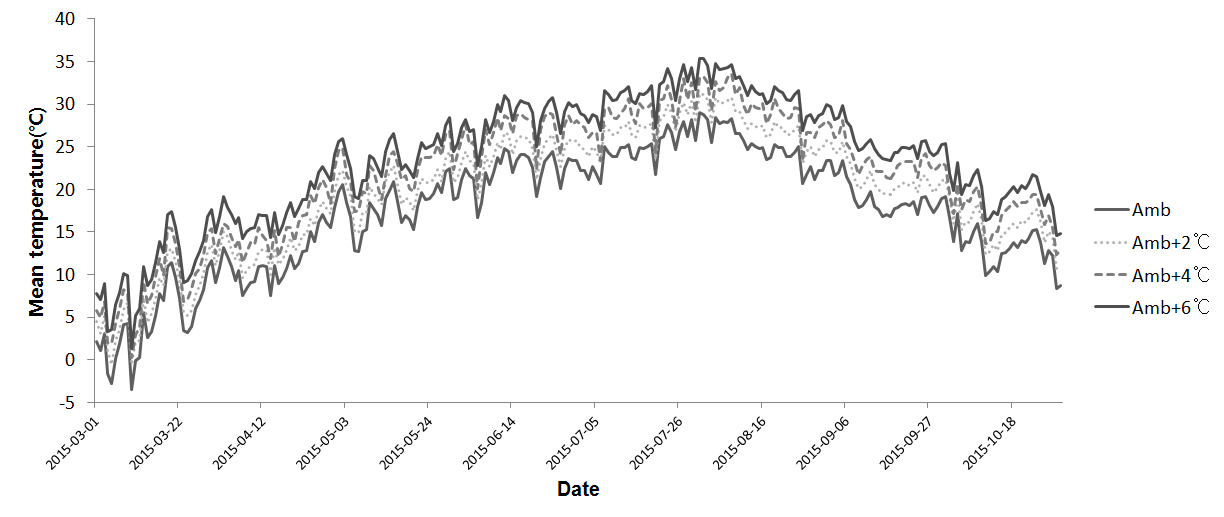


Figure S1. Changes in daily average air temperature in the Temperature Gradient Greenhouse where Korean ginseng, Yunpoong, grown. Each label represents a location within the temperature-gradient green house (TGG), which have different temperature measures. Amb, Ambient Temperature; Amb+2℃, Ambient Temperature +2
