# Supplementary material for: The mRNA and miRNA transcriptomic landscape of Panax ginseng under the high ambient temperature
Source: BMC Syst Biol. 2018 Mar 19;12(Suppl 2):27. doi: 10.1186/s12918-018-0548-z (PMC5861484; doi:10.1186/s12918-018-0548-z)
Supplement: Supplementary file 6 — Results related to sanger sequencing of asssembled contigs. The primer sequences, sanger sequence of selected contigs and BLAST results on NR and UniRef90 databases are shown. (DOC 733 kb) [file 12918_2018_548_MOESM6_ESM.docx]

**Additional File 6**

A total of 5 synthesized sequences corresponding to the following contigs are shown along with their BLAST results on the corresponding contigs:

- Sequence 1: TRINITY_DN215770_c7_g28_i1 (R3H)
- Sequence 2: Contig8260 (R3H)
- Sequence 3: Contig2268 (MET1)
- Sequence 4: TRINITY_DN215566_c4_g6_i1 (DM1)
- Sequence 5: TRINITY_DN169002_c1_g1_i1 (Chlorophyll a-b)

For each of the five genes, a full sequence primer has been synthesized based on the target contig sequence, which has been BLASTX searched in the Arabidopsis thaliana plant protein sequence database (TAIR10). For the full sequences with a clear band, sanger sequence has been performed to synthesize the sequence between the forward and reverse primers. The sequence of the sanger sequencing result has been searched in our assembled contig sequence database to validate its presence in our RNA-seq sample. At last, BLASTX search results of the contigs on the Arabidopsis database TAIR10 is shown.

**Sequence 1**

Target gene = Single-stranded nucleic acid binding R3H protein

- **Full sequence (1,406bp) primer**

|  | TAAGAAGAGGGGGGGCAAGAAAAAGACAAAAATAAAATTAGATCTAGATAGCCCTATTTTTCTCTCTCCACGGTTTTTTTTGGGAGGCGATAAGAAGAGGGAGTGGGGGAGAAAAACAGAGTTTTCTCTCTTAAACCTTCCCTTTCGGGAGGAGAGAGAAGAAGAAGATACTCCCCTACTTTCCTCTTTTCTTTTCCCACCAGAAGAGAGAAGAAGAGGAGAAACAAACACAATCCCTATATATAGGATTATATATTTAGAGAGAGAAAGTTAGAGAGAGAGGGGGCTTTCTCTCTCCACAATGGACTCTTCATCACCGCCACAATCAACCACCGTCGCCGATGATTCCCTCGCCGTTAATGCACCGATGGTGGATCCCTTCTTGGTCGAGGCTCTCCAAAACCCTCGTCATCGTTTCACCATTCTGCGAATGGAACTCGATATTCAAAAGTTTTTACAGAACTCTGATCAACAACAGTTTGAGTTCCAACATTTCCCAACTTCTTACCTTCGTCTTGCAGCACACCGAGTTGCTCAACACTATGGTCTGCCAACTATGGTTCAGGATAATTTGGTTGATGGCCAGGGAACCAGAATTGTGGTCAGAAAAATAGCTGAAAGTATTTACCCTTCTATTTGTTTGTTGGAGATACCAGTTAAACAGTCTGAAAATGACAAGCTTGAGCAGAAAAAAATTGTCATAAGACCAAGGCCTAGTAAAGCCTCCTTAAATGGAACCAGTGAATTAGGGAAGCGAAGTTCCATAAGAACTGTGGAAGAGAGGAAGGAGGAGTACGACAGGGCACGAGCTCGGATATTCAGTAGTCCTGAGTCAGAAGATACAGTTTCTCTGGTCCCTGTCGATGGGAAGAATGCATATTTTACCGGGGATGAAAATGAAGGTTCCAGGAATGCTGTCACGGACTTGGAGAGAACTTCAGTGGCAGGGATGTTAGTGCTGCTTCACGAGTAGCCATTTTCAGAGATAGGGAGAAAGATCGCACAGATCCGGATTATGATCGGAGTTACGATAGGTATGTCAGAAATCTTCCTAGCAGTCAAAACTTCAACTTGACGCCTTTTAATATGCAGAAGTTTCAGCCTCCGTATCTGCAGTATGATTCTGTTTTTCCTCAGTTGGGACAGGTGCCAATCACTCAAGCTTCACTTGGCTACAGGAGCCCTATCATGAGCCCTTACTGTGCCATTGGACCGAATCAGACATCAAGGGATCCTGTCTTTATGCAGTGGCCATCTCATGCTATGATGTACACACAGTCATATGATCAGTTTAGACATGCTGTTTTTCAGGCTCCATTTTGTCAGCAGCCCCTGAGTTTTGATTACTCCCAAAACCACTGAATATCAACTAGTAGGCATCAGAGGGAACTGTCCTTTCACTCAGAACTAAGTCATTATTTTAATTTAGGGTTCTCCTTTTAGGTCTCATTGTCAAAGAAGAATTATTTTTCTTGTTGAACTTGTGTGTTACTTTCCTTGTTTTCACATTTGTTGGTATGATTTGGTAAGGGGGGAGGGATGACATATATTAGTATAATTAAATCCAAGTACTATTATTGGTTTTGGCGTGACCCTGTTTATTTTGTTTAGTCCTATGCGGACATGTAAATTGGATAGGAAGAACAAAAGGAATTTGTTCTCGCGAACAATTTGACATTACAATTCATTTGTTTGCCAGGACAAATTTCTTTTGTTCTTTCGGTCCAATTATTCTGTTTAATTTACCCGTCTGTAAAGAGTTATTGTTTTGTTTATGGTTCTCATGTTTTTTCCATCCTGGGTCTTATGACTCACATAAAAAAGAGTCGAATAATGTTCATATACACATGTATACTGCGATCTTTTTTTATTTGATAGTAGCATATGTTGCGGATAATGATTAATGATGCATCTTATTGAATGTACGGGTTTATATATGCATGCCGTCAGTCATACTGGTAATACAAAAAATTGTAGACTCCCCAACTAGCCCATTCCCCAGAACAAACCAAGAAATTGCAAACATTTTTTTTATGTATTAAAAAAGATCTATAGTTAGAATTATATTTATCAGGAGAATTTTATACCTTTTTCAAAAGTCAACGACCACTTTCGGCACTAGGGCACAACGAAAACACTCCGTTTTCCCTTTGTACCAAGTCACCAACTTTAAGAAAATTAACACCTTGTTGCCCCACGCTCAATGTAAGTGCTAATTTGGTGCTGTTTTAAGGTTTAAAAGACCCAAAAAAAGAAGAAGTGCTTTATAGAATTGTCACAAGTAGAAGAAGTCCTTTTTGACATAGAAAAAGGTCTGTAATCTGGAAGGTTAGTTTCAGATTTTGTGCAGCCCTCCAAAGGAAATACAAACCCACATGGGGTCGGTTTATTATGGTATGGGCAGTCATGACCTGGAACACATTAGCTCTAATTTTATATCAATTTTCTTTCTATACACTTACATTTTACATGGACAATGGAGGCTTCATTAATCTGTATTATAATATTGAGCTAAACATAGAAATCATAATTTAATCAAAGATACATGGATACTCGAGTATCTAAAGTCTACACAGATCAGATCTAACCCAACCCAACATAATACAATACAAAATTAGTTGATGATTAAAAATGAAGGGATCTAAAAGTAAATTAATCAATGCAATCAAATACATGCTAATCTGACAGTTCATTGCATGTAGGGGGGGCATGCAAGTTGTTTCAAGTCCAGTTTTATGGTTCCTGTGGAGCTGACATTATACTAAATGGGATTCTATACTTTGTCGCCAAATACGTGTCACTATTATATTGGTGGCTTAATAATGGCATGTGTATTTGGGACACAAACTTTAGGGATATAAAATGGAATCCCTAGCACAACTCCACATGATAACTTACACAAATTATCTTTATTATGTTACAATTTGTTTAAAGACTGTAACCTTGTCAACAATGAAAGATGATCTCTATCCATCATGCAGGCAATATCATCAACCAATGTGGGATCAAAATCCAGTTGTCATGAAGCTGTTTATGAGTTGGAACATTATTGCAGATGTTGCAGCTTCAGTATCCAAAGAAGGGAAGTATCCATATTCCAATTAGATTCAGTCATCCCCTGTTGGAAACAGAGTGAGAACTTTCCTTCCATTGGCTGCTGCTGCTGCTGTTGCGGGTTCCAAATCTTCAACATAAGAAGATTGCACTGAATTATGCACAGGTGTCCCCATCCTATGAACCTGCTGCTGCTGTGATTGTCTTGCCAGAAACGGATGCTCATATTGCCCATTTGGGGGAGACCCAAAATGCCCCACGTGCCGATACTGCTGATGTCCGGCAACAGCTGCCGCGGCCGCCATCTGCTGCTGATGATGGTGCTGCTGCAGCGCAGCAACAGGCACAAACGGCAAGAAATGGTCAGAATTCGGACGCCCAGGGTGCAGGAAATGAGCCGGCACAGGTGAACTGGCAAACAAATGATCCGTGGCGGGGTCCACTGCAGAAGAAAGACCTCCGCCACCACCGCCTCCTCCGGCGGAAAGCCCCTGCATCCGCTTCAGGTAAAGTCTGTACTTTTGCAAATGACTGGCAACGTTCTCACGGGTCAACCCATCCACACTCATGAGCTGCATTATGGTCTTGGGGACCGCGTTCTTGATCCCCAGGTGGCCCACAGCATCCACAAATCTTTTGTGTAGCTGCGGTGTCCACACGAGGCGGGGCCGCTTCAGGGTGCGGGCCGGCTCGTCTCCCGCAGCACCGCCACCCAACTCAGAGGAATCAAACTCGGCGGAATTGGGCTGCGAGGAAGGTGTCTGAACCGGTGGTAACGGTGGCGGAGGTGGTGGGTTTTGTTGTTGGGCTTGGTGAAGATTATTGTTTGGGTTTCGAATATCGAAGGCTAAAGCAAGATCAGGGGTTATTAGGGTTTGAGATAAGGGCATGAGCTCATCAGGGGAAGGAAGTTCTTCTTCCCACTTTGAAAACCAATTTGACTCATCTTCTCTCATTTCTTTTACCACTTTTGATCAAAAGTATTTCTTCCCTTTAAGAGTGGTAAAATTGAAAGTATATATTTTTGTGGGTCAAGATTTCTGTATCAATTAATTATTTAACAGAAACAAAGAAGAAGAAGAAGCAAAGAGGCAGATGAGTTGAGTATGACAAAATCAAAGACGGATGGAAATTGGTAGTAGATGGCTGGCTTTGTTATGTTATCTCTCCCCCTCTCTCTCTAGAAAGAAGGAAAGACTGAATTTTTTAGAGAGAGGATAGGGGAGGGAGAATGGAGTCTGGAGGTTTTTGGGAGTGGGGTGGAAATGGAGGTGGAGGGGCCAATCTTGCGTATTCAAATGCAATAAGCCTTTT |
| --- | --- |

**Lambda/Hind iii cut**

**1kb ladder**

**R3H (set2)**

**PCK1 (set2)**

**FD1 (set2)**

**PCK1 (set1)**

**R3H (set1)**

**FD1 (set1)**

**Lambda/Hind iii cut**


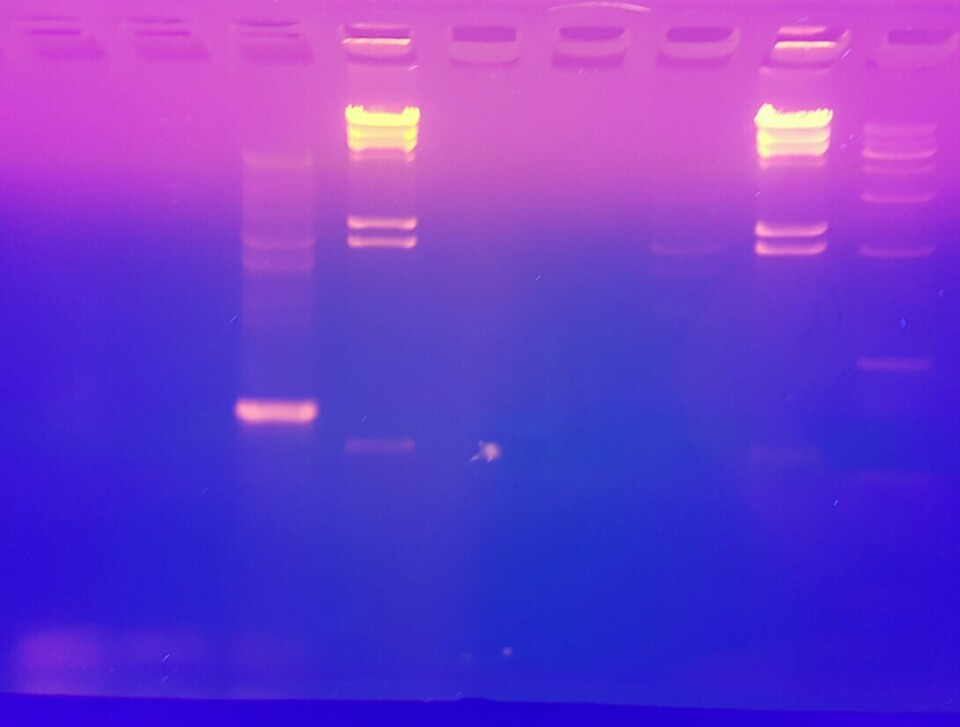


- **R3H Sanger Sequencing result**

>170302-059_G03_plasmid_5_primer_3.ab1 [626bp]

GCTGAACGTCTGCGATGGGTCTCTATTTCAAAGTTTTTACAGAACTCTGATCAACAACAGTTTGAGTTCCAACATTTCCCAACTTCTTACCTTCGTCTTGCAGCACACCGAGTTGCTCAACACTATGGTCTGCCAACTATGGTTCAGGATAATTTGGTTGATGGCCAGGGAACCAGAATTGTGGTCAGAAAAATAGCTGAAAGTATTTACCCTTCTATTTGTTTGTTGGAGATACCAGTTAAACAGTCTGAAAATGACAAGCTTGAGCAGAAAAAAATTGTCATAAGACCAAGGCCTAGTAAAGCCTCCTTAAATGGAACCAGTGAATTAGGGAAGCGAAGTTCCATAAGAACTGTGGAAGAGAGGAAGGAGGAGTACGACAGGGCACGAGCTCGGATATTCAGTAGTCCTGAGTCAGAAGATACAGTTTCTCTGGTCCCTGTCGATGGGAAGAATGCAAATTTTGCCGGGGATGAAAATGAAGGTTCCGGGAGTGCTGTCACGGACTTGGAGAAAAACTTCAGAGGCAGGGATGTTAGTGCTGCTTCACGAGTATCCATTTTCAGAGATATGGAGAAAGATCACAAAGATCCGGATTATGATCAGAGTTACCATAGGTATGTTAG

>170302-059_I03_plasmid_5_primer_4.ab1 [637bp]

GCATTGAAGAAATGGACTGGCTAGCTTGCTTTCTGACATACCTATCGTAACTCCGATCATAATCCGGATCTGTGCGATCTTTCTCCCTATCTCTGAAAATGGCTACTCGTGAAGCAGCACTAACATCCCTGCCACTGAAGTTTCTCTCCAAGTCCGTGACAGCATTCCTGGAACCTTCATTTTCATCCCCGGTAAAATATGCATTCTTCCCATCGACAGGGACCAGAGAAACTGTATCTTCTGACTCAGGACTACTGAATATCCTAGCTCTTGCCCTGTCGGACTCCTCCTTCCCCTCTTCCACAGTTCTCATGGAACTTCGGTTCCCTAATTCACTGGTTCCATTTAAGGAGGCTTTAGGAGGCCTAGGTCTTATGGGAATTTTTTTCGGCTCAAGCTGGTCAATTTCGTACTGTTTAAATGGTATATCCGACAAACAAATAAAAGGGTAAATACTTTCAGCTATTTTTCTGACCACAATTCTGGTTCCTTGGGCATCTTCCAAATTATCCTGAACCATATTTGGCAGACCATAGTGTTGAGCAACTCGATGTGCTGCATGACGAACGTAAGAAGTTGGGAAATGTTGGAACTCTTACTGTTGTTGATGATAGTTCTGAAAAAACTTTTGAAATAT

**▶ BlastN result of the Full sequence and the synthesized sanger sequence (170302-059_G03_plasmid_5_primer_3)**

full TGCACCGATGGTGGATCCCTTCTTGGTCGAGGCTCTCCAAAACCCTCGTCATCGTTTCAC 420

forward ----------------------------------------------------------GC 2

*

full CATTCTGCGAATGGAACTCGATATTCAAAAGTTTTTACAGAACTCTGATCAACAACAGTT 480

forward TGAACGTCTGCGATGGGTCTCTATTTCAAAGTTTTTACAGAACTCTGATCAACAACAGTT 62

* * ** **** *********************************

full TGAGTTCCAACATTTCCCAACTTCTTACCTTCGTCTTGCAGCACACCGAGTTGCTCAACA 540

forward TGAGTTCCAACATTTCCCAACTTCTTACCTTCGTCTTGCAGCACACCGAGTTGCTCAACA 122

************************************************************

full CTATGGTCTGCCAACTATGGTTCAGGATAATTTGGTTGATGGCCAGGGAACCAGAATTGT 600

forward CTATGGTCTGCCAACTATGGTTCAGGATAATTTGGTTGATGGCCAGGGAACCAGAATTGT 182

************************************************************

full GGTCAGAAAAATAGCTGAAAGTATTTACCCTTCTATTTGTTTGTTGGAGATACCAGTTAA 660

forward GGTCAGAAAAATAGCTGAAAGTATTTACCCTTCTATTTGTTTGTTGGAGATACCAGTTAA 242

************************************************************

full ACAGTCTGAAAATGACAAGCTTGAGCAGAAAAAAATTGTCATAAGACCAAGGCCTAGTAA 720

forward ACAGTCTGAAAATGACAAGCTTGAGCAGAAAAAAATTGTCATAAGACCAAGGCCTAGTAA 302

************************************************************

full AGCCTCCTTAAATGGAACCAGTGAATTAGGGAAGCGAAGTTCCATAAGAACTGTGGAAGA 780

forward AGCCTCCTTAAATGGAACCAGTGAATTAGGGAAGCGAAGTTCCATAAGAACTGTGGAAGA 362

************************************************************

full GAGGAAGGAGGAGTACGACAGGGCACGAGCTCGGATATTCAGTAGTCCTGAGTCAGAAGA 840

forward GAGGAAGGAGGAGTACGACAGGGCACGAGCTCGGATATTCAGTAGTCCTGAGTCAGAAGA 422

************************************************************

full TACAGTTTCTCTGGTCCCTGTCGATGGGAAGAATGCATATTTTACCGGGGATGAAAATGA 900

forward TACAGTTTCTCTGGTCCCTGTCGATGGGAAGAATGCAAATTTTGCCGGGGATGAAAATGA 482

************************************* ***** ****************

full AGGTTCCAGGAATGCTGTCACGGACTTGGAGAG-AACTTCAGTGGCAGGGATGTTAGTGC 959

forward AGGTTCCGGGAGTGCTGTCACGGACTTGGAGAAAAACTTCAGAGGCAGGGATGTTAGTGC 542

******* *** ******************** ******** *****************

full TGCTTCACGAGTAGCCATTTTCAGAGATAGGGAGAAAGATCGCACAGATCCGGATTATGA 1019

forward TGCTTCACGAGTATCCATTTTCAGAGATATGGAGAAAGATCACAAAGATCCGGATTATGA 602

************* *************** *********** ** ***************

full TCGGAGTTACGATAGGTATGTCAGAAATCTTCCTAGCAGTCAAAACTTCAACTTGACGCC 1079

forward TCAGAGTTACCATAGGTATGTTAG------------------------------------ 626

** ******* ********** **

full TTTTAATATGCAGAAGTTTCAGCCTCCGTATCTGCAGTATGATTCTGTTTTTCCTCAGTT 1139

forward ------------------------------------------------------------ 626

**▶ BlastN result of the Full sequence and the synthesized sanger sequence (170302-059_I03_plasmid_5_primer_4_reverse complement seq)**

full TGCACCGATGGTGGATCCCTTCTTGGTCGAGGCTCTCCAAAACCCTCGTCATCGTTTCAC 420

reverse ------------------------------------------------------------ 0

full CATTCTGCGAATGGAACTCGATATTCAAAAGTTTTTACAGAACTCTGATCAACAACAGTT 480

reverse -------------------ATATTTCAAAAGTTTTTTCAGAACTATCATCAACAACAGTA 41

************* ******* * ************

full TGAGTTCCAACATTTCCCAACTTCTTACCTTCGTCTTGCAGCACACCGAGTTGCTCAACA 540

reverse AGAGTTCCAACATTTCCCAACTTCTTACGTTCGTCATGCAGCACATCGAGTTGCTCAACA 101

*************************** ****** ********* **************

full CTATGGTCTGCCAACTATGGTTCAGGATAATTTGGTTGATGGCCAGGGAACCAGAATTGT 600

reverse CTATGGTCTGCCAAATATGGTTCAGGATAATTTGGAAGATGCCCAAGGAACCAGAATTGT 161

************** ******************** **** *** **************

full GGTCAGAAAAATAGCTGAAAGTATTTACCCTTCTATTTGTTTGTTGGAGATACCAGTTAA 660

reverse GGTCAGAAAAATAGCTGAAAGTATTTACCCTTTTATTTGTTTGTCGGATATACCATTTAA 221

******************************** *********** *** ****** ****

full ACAGTCTGAAAATGACAAGCTTGAGCAGAAAAAAATTGTCATAAGACCAAGGCCTAGTAA 720

reverse ACAGTACGAAATTGACCAGCTTGAGCCGAAAAAAATTCCCATAAGACCTAGGCCTCCTAA 281

***** **** **** ********* ********** ********* ****** ***

full AGCCTCCTTAAATGGAACCAGTGAATTAGGGAAGCGAAGTTCCATAAGAACTGTGGAAGA 780

reverse AGCCTCCTTAAATGGAACCAGTGAATTAGGGAACCGAAGTTCCATGAGAACTGTGGAAGA 341

********************************* *********** **************

full GAGGAAGGAGGAGTACGACAGGGCACGAGCTCGGATATTCAGTAGTCCTGAGTCAGAAGA 840

reverse GGGGAAGGAGGAGTCCGACAGGGCAAGAGCTAGGATATTCAGTAGTCCTGAGTCAGAAGA 401

* ************ ********** ***** ****************************

full TACAGTTTCTCTGGTCCCTGTCGATGGGAAGAATGCATATTTTACCGGGGATGAAAATGA 900

reverse TACAGTTTCTCTGGTCCCTGTCGATGGGAAGAATGCATATTTTACCGGGGATGAAAATGA 461

************************************************************

full AGGTTCCAGGAATGCTGTCACGGACTTGGAGAGA-ACTTCAGTGGCAGGGATGTTAGTGC 959

reverse AGGTTCCAGGAATGCTGTCACGGACTTGGAGAGAAACTTCAGTGGCAGGGATGTTAGTGC 521

********************************** *************************

full TGCTTCACGAGTAGCCATTTTCAGAGATAGGGAGAAAGATCGCACAGATCCGGATTATGA 1019

reverse TGCTTCACGAGTAGCCATTTTCAGAGATAGGGAGAAAGATCGCACAGATCCGGATTATGA 581

************************************************************

full TCGGAGTTACGATAGGTATGTCAGAAATCTTCCTAGCAGTCAAAACTTCAACTTGACGCC 1079

reverse TCGGAGTTACGATAGGTATGTCAGAAAGCAAGCTAGCCAGTCCATTTCTTCAATGC---- 637

*************************** * ***** * * **

full TTTTAATATGCAGAAGTTTCAGCCTCCGTATCTGCAGTATGATTCTGTTTTTCCTCAGTT 1139

reverse ------------------------------------------------------------ 637

**BlastN search result of Sequence 1 (170302-059_G03_plasmid_5_primer_3) on the assembled contigs**

> TRINITY_DN215770_c7_g28_i1 (g.8872_[Putative uncharacterized protein]와 g.8873[Homeodomain-like superfamily protein])

Length=4369

Score = 1038 bits (562), Expect = 0.0

Identities = 600/618 (97%), Gaps = 3/618 (0%)

Strand=Plus/Plus

Query 9 TCTGCG-ATGGGTCTCTAT-TTC-AAAGTTTTTACAGAACTCTGATCAACAACAGTTTGA 65

|||||| |||| ||| || ||| ||||||||||||||||||||||||||||||||||||

Sbjct 424 TCTGCGAATGGAACTCGATATTCAAAAGTTTTTACAGAACTCTGATCAACAACAGTTTGA 483

Query 66 GTTCCAACATTTCCCAACTTCTTACCTTCGTCTTGCAGCACACCGAGTTGCTCAACACTA 125

||||||||||||||||||||||||||||||||||||||||||||||||||||||||||||

Sbjct 484 GTTCCAACATTTCCCAACTTCTTACCTTCGTCTTGCAGCACACCGAGTTGCTCAACACTA 543

Query 126 TGGTCTGCCAACTATGGTTCAGGATAATTTGGTTGATGGCCAGGGAACCAGAATTGTGGT 185

||||||||||||||||||||||||||||||||||||||||||||||||||||||||||||

Sbjct 544 TGGTCTGCCAACTATGGTTCAGGATAATTTGGTTGATGGCCAGGGAACCAGAATTGTGGT 603

Query 186 CAGAAAAATAGCTGAAAGTATTTACCCTTCTATTTGTTTGTTGGAGATACCAGTTAAACA 245

||||||||||||||||||||||||||||||||||||||||||||||||||||||||||||

Sbjct 604 CAGAAAAATAGCTGAAAGTATTTACCCTTCTATTTGTTTGTTGGAGATACCAGTTAAACA 663

Query 246 GTCTGAAAATGACAAGCTTGAGCAGaaaaaaaTTGTCATAAGACCAAGGCCTAGTAAAGC 305

||||||||||||||||||||||||||||||||||||||||||||||||||||||||||||

Sbjct 664 GTCTGAAAATGACAAGCTTGAGCAGAAAAAAATTGTCATAAGACCAAGGCCTAGTAAAGC 723

Query 306 CTCCTTAAATGGAACCAGTGAATTAGGGAAGCGAAGTTCCATAAGAACTGTGGAAGAGAG 365

||||||||||||||||||||||||||||||||||||||||||||||||||||||||||||

Sbjct 724 CTCCTTAAATGGAACCAGTGAATTAGGGAAGCGAAGTTCCATAAGAACTGTGGAAGAGAG 783

Query 366 GAAGGAGGAGTACGACAGGGCACGAGCTCGGATATTCAGTAGTCCTGAGTCAGAAGATAC 425

||||||||||||||||||||||||||||||||||||||||||||||||||||||||||||

Sbjct 784 GAAGGAGGAGTACGACAGGGCACGAGCTCGGATATTCAGTAGTCCTGAGTCAGAAGATAC 843

Query 426 AGTTTCTCTGGTCCCTGTCGATGGGAAGAATGCAAATTTTGCCGGGGATGAAAATGAAGG 485

|||||||||||||||||||||||||||||||||| ||||| |||||||||||||||||||

Sbjct 844 AGTTTCTCTGGTCCCTGTCGATGGGAAGAATGCATATTTTACCGGGGATGAAAATGAAGG 903

Query 486 TTCCGGGAGTGCTGTCACGGACTTGGAGAAAAACTTCAGAGGCAGGGATGTTAGTGCTGC 545

|||| ||| |||||||||||||||||||| ||||||||| ||||||||||||||||||||

Sbjct 904 TTCCAGGAATGCTGTCACGGACTTGGAGAGAAACTTCAGTGGCAGGGATGTTAGTGCTGC 963

Query 546 TTCACGAGTATCCATTTTCAGAGATATGGAGAAAGATCACAAAGATCCGGATTATGATCA 605

|||||||||| ||||||||||||||| ||||||||||| || |||||||||||||||||

Sbjct 964 TTCACGAGTAGCCATTTTCAGAGATAGGGAGAAAGATCGCACAGATCCGGATTATGATCG 1023

Query 606 GAGTTACCATAGGTATGT 623

||||||| ||||||||||

Sbjct 1024 GAGTTACGATAGGTATGT 1041

**▶BLASTX search result of TRINITY_DN215770_c7_g28_i1 and R3H (AT3G56680)**

>[AT3G56680](http://www.arabidopsis.org/servlets/TairObject?type=locus&name=AT3G56680).1 | Symbols: | Single-stranded nucleic acid binding R3H protein |

chr3:20991537-20993446 FORWARD LENGTH=353

Length = 353

Score = 346 bits (887), Expect = 9e-95

Identities = 188/337 (55%), Positives = 231/337 (68%), Gaps = 8/337 (2%)

Frame = +2

Query: 371 VDPFLVEALQNPRHRFTILRMELDIQKFLQNSDQQQFEFQHFPTSYLRLAAHRVAQHYGL 550

VDPFLVEAL N RHR TILRMELD+Q+ LQN +QQQFEFQHFPTSYLRLAAHRVA HYGL

Sbjct: 24 VDPFLVEALHNSRHRLTILRMELDVQRLLQNPEQQQFEFQHFPTSYLRLAAHRVANHYGL 83

Query: 551 PTMVQDNLVDGQGTRIVVRKIAESIYPSICLLEIPV-KQSENDKLEQKKIVIRPRPSKAS 727

T VQ++ DG RI+V K ES +P++ L EIPV KQSEN K E +K+ I+ RPSK S

Sbjct: 84 ATAVQESGADGNENRILVTKTTESKFPAVRLSEIPVAKQSENGKFESRKVSIKTRPSKGS 143

Query: 728 LNGTSELGK-RSSIRTVEERKEEYDRARARIFS---SPESEDTVSLVPVDGKNAYFTGDE 895

G +L K R +R+VEERKEEYD+AR RIFS +D+ S V +NA + D+

Sbjct: 144 GYGAGDLEKNRGPLRSVEERKEEYDKARERIFSGLTGLSCDDSSSETQVYERNASLSRDD 203

Query: 896 NEGSRNAVTDLERNFSGRDVSAASRVAIFRDREKDRTDPDYDRSYDRYVRNLPSSQNFNL 1075

+ S+NA ++++N S R+ SRVAIFRDREKDR DPDYDR + RY+R+LP +QNFNL

Sbjct: 204 KQVSKNAYVEVKKNLSIRESGPTSRVAIFRDREKDRFDPDYDRRHQRYIRSLPVNQNFNL 263

Query: 1076 TPFNMQKFQPPYLQYDSVFPQLGQVPITQASLGY---RSPIMSPYCAIGPNQTSRDPVFM 1246

PFN+Q+ PY Y+ F Q+P A LG+ S IMSPY T+ D ++M

Sbjct: 264 PPFNIQQIPTPY--YEMGFTGYNQIPSPPAPLGFGPHPSSIMSPY------GTTMDAMYM 315

Query: 1247 QWPSHAMMYTQSYDQFRHAVFQAPFCQQPLSFDYSQN 1357

WP+ AMMY Y+QFR+ QA F QQPLSFDY QN

Sbjct: 316 HWPNAAMMYAHPYEQFRNGSLQAQFVQQPLSFDYMQN 352

**Sequence 2**

Target gene = Single-stranded nucleic acid binding R3H protein

- **R3H Sanger Sequencing result**

>170228-071_A09_plasmid_5_primer_3.ab1 665

GGCATCGGTCTGCGATGGATTCGATATTCAAAGTTTTTACAGAACTCTGATCAACAACAGTTTGAGTTCCAACATTTCCCAACTTCTTACCTTCGTCTTGCAGCACACCGAGTTGCTCAACACTATGGTCTGCCAACTATGGTTCAGGATAATTTGGTTGATGGCCAGGGAACCAGAATTGTGGTCAGAAAAATAGCTGAAAGTATTTACCCTTCTATTTGTTTGTTGGAGATACCAGTTAAACAGTCTGAAAATGACAAGCTTGAGCAGAAAAAAATTGTCATAAGACCAAGGCCTAGTAAAGCCTCCTTAAATGGAACCAGTGAATTAGGGAAGCGAAGTTCCATAAGAACTGTGGAAGAGAGGAAGGAGGAGTACGACTGGGCACGAGCTCGTATATTCAGTAGTCCTGAGTCGGAAGATACAGATTCTCCGGTCCCTGTCGATGGGATGAATGCATATTTTGCCAGGGATGAAAATGAAGGTTCCGGGAGTGCTGTCACAGCCTTAAAGAGAATCGTCAGAGGCTGCGATGTTAGGTGCTGCTTCTGCAGCATCCACGATTCAGAGATAGTGGAAAAAAGATCGGAAAGATCCGGATTATGAGCAGAATTTTCTGTAGATAATCGTTTGGAATCTTCCGAAGCATTCAAAACAATCGACGT

**BlastN search result of Sequence 2 on the assembled contigs**

Query= 170228-071_A09_plasmid_5_primer_3.ab1 665

Length=665

Sequences producing significant alignments: Score (Bits) E Value

Contig8260 268 4e-71

Length=4369

Score = 898 bits (486), Expect = 0.0

Identities = 564/601 (94%), Gaps = 7/601 (1%)

> Contig8260

Length=482

Score = 268 bits (145), Expect = 4e-71

Identities = 323/408 (79%), Gaps = 16/408 (4%)

Query 208 TACCCTTCTATTTGTTTGTTGGAGATACCAGTTAAACAGTCTGAAAATGACAAGCTTGAG 267

|||||| || | |||||||| || |||||| |||| |||| ||||||||||| ||||||

Sbjct 1 TACCCTGCTGTGTGTTTGTTTGATGTACCAGGTAAAGAGTCAGAAAATGACAAACTTGAG 60

Query 268 CAGaaaaaaaTTGTCATAAGACCAAGGCCTAGTAAAGCCTCCTTAAATGGAACCAGTGAA 327

||||||||||||||||| ||||||||||||| || | || || |||||||||||||||

Sbjct 61 CAGAAAAAAATTGTCATTAGACCAAGGCCTA--AA-GACTACTCAAATGGAACCAGTGAG 117

Query 328 TTAGGGAAGCGAAGTTCCATAAGAACTGTGGAAGAGAGGAAGGAGGAGTACGACTGGGCA 387

|| ||||| ||| |||||||||||||||||||||||||||||| ||||||||| |||||

Sbjct 118 TTGGGGAAAAGAAATTCCATAAGAACTGTGGAAGAGAGGAAGGAAGAGTACGACAGGGCA 177

Query 388 CGAGCTCGTATATTCAGTAGTCCTGA--GTCG-GA---AGA-TA--CAGATTCTCCGGTC 438

|||||||||||||| ||| ||||| | ||| || ||| || ||| |||| || |

Sbjct 178 CGAGCTCGTATATTTAGTGGTCCTAACAGTCCCGAGTCAGAATATACAGTGTCTCAGGCC 237

Query 439 CCTGTCGATGGGATGAATGCATATTTTGCCAGGGATGAAAATGAAGGTTCCGGGAGTGCT 498

| ||| ||||||| ||||| ||| | | | ||||||||||||| ||| || | || | ||

Sbjct 238 CTTGTAGATGGGAAGAATGTATACTATTCTAGGGATGAAAATGTAGGGTCAGTGAATACT 297

Query 499 GTCACAGCCTTAAAGAGAATCGTCAGAGGCTGCGATGTTAGGTGCTGCTTCTGCAGCATC 558

|||| | | | |||||| | |||| ||| | |||| | ||| || |||| || | |

Sbjct 298 GTCATGGACATGGAGAGAAACTTCAGTGGCAGGGATGGT-GGTACTTCTTCACGAGTAGC 356

Query 559 CACGATTCAGAGATAGTGGAAAAAAGATCGGAAAGATCCGGATTATGA 606

|| ||||||||||| ||| || ||||| | |||||| ||||||||

Sbjct 357 CATT-TTCAGAGATAG-GGAGAAG-GATCGCACAGATCCTGATTATGA 401

**▶BLASTX search result of Contig8260 and R3H (AT3G56680)**

>[AT3G56680](http://www.arabidopsis.org/servlets/TairObject?type=locus&name=AT3G56680).1 | Symbols: | Single-stranded nucleic acid binding R3H protein |

chr3:20991537-20993446 FORWARD LENGTH=353

Length = 353

Score = 127 bits (320), Expect = 3e-30

Identities = 70/151 (46%), Positives = 98/151 (64%), Gaps = 3/151 (1%)

Frame = +1

Query: 1 YPAVCLFDVP-GKESENDKLEQKKIVIRPRPKDYSN-GTSELGK-RNSIRTVEERKEEYD 171

+PAV L ++P K+SEN K E +K+ I+ RP S G +L K R +R+VEERKEEYD

Sbjct: 109 FPAVRLSEIPVAKQSENGKFESRKVSIKTRPSKGSGYGAGDLEKNRGPLRSVEERKEEYD 168

Query: 172 RARARIFSGPNSPESEYTVSQALVDGKNVYYSRDENVGSVNTVMDMERNFSGRDGGTSSR 351

+AR RIFSG + + S+ V +N SRD+ S N +++++N S R+ G +SR

Sbjct: 169 KARERIFSGLTGLSCDDSSSETQVYERNASLSRDDKQVSKNAYVEVKKNLSIRESGPTSR 228

Query: 352 VAIFRDREKDRTDPDYDRSYDRYVLCIEIGE 444

VAIFRDREKDR DPDYDR + RY+ + + +

Sbjct: 229 VAIFRDREKDRFDPDYDRRHQRYIRSLPVNQ 259

**Sequence 3**

Target gene = MET1 gene


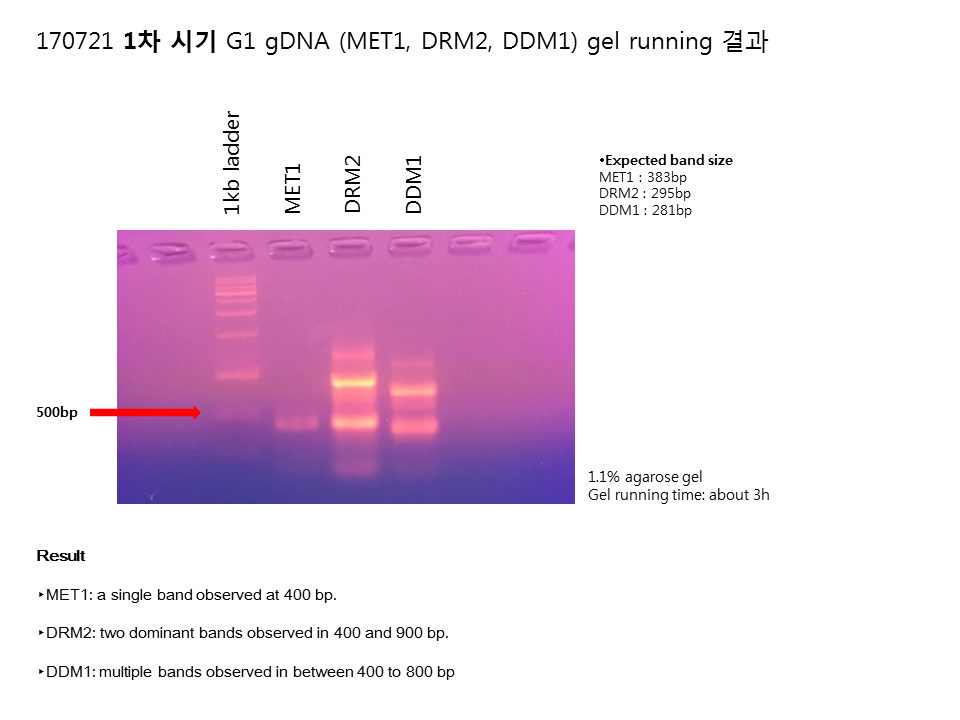


- **MET1 Sanger Sequencing result**

**Forward sequence (Sanger sequence)**

>170804-041_C03_20170804M_20170717G1.ab1 484
AGGGTGCTGTTTTAGGGATTCAAGGTGTCTGGGTTCGACCTGATAACAAAGTTGAATGGGATCCTGATGTAGAACGCGTGTATCTGCCTTCAGGAAAGCCACTGGTACCTGATTATGCAATGACTTTTGTCGGAGGAACTTCTTCGAAACCATTTAGTCGGTTATGGTGGGATGAAACTGTACCTACTGTAGTTACCAGGGCTGAACCCCATAATCAGACTATCACACATCCTGAACAAAACAGGGTACTCACAATCCGTGAGAATGCCAGACTTCAAGGATTTCCAGATTATTATCAGCTCGAAAGCCCCAATAAATAAGGTTTAGTTAAATTGTGTTGGTGTGGTGGGTCGTTGGTGGTGTGGTGGGTTGTGGGGGGGGTTGTGTGAGGTTGTTGGTTGGGGGGGTTTTGGGGGGTGGTGTGGCAGGTTTTGGGGTGGGTTGGGGTTTGTGGGGGGGGGGTGGGTGGTGGGGTGTTTTTGGG

**Reverse complement sequence (Sanger sequence)**

>170804-041_E03_20170804M_20170801G11.ab1 501
CACCCCCCCCCGACCCAGAAGCTATAACCCTCCTACACCTACCCCTTAGGCAAGAACATAGGCCCACCCCCCCCCCCCATCCCCACCGCACCTCATCCGAAACCCCCCCCCCACTACCTTCCCGCCACCCCGCCCACTCTCTACCCCCCCCCCCCCCCCCCTCATCTTCTCCCCCCCATTTTTCAACGTTTTTTTCCATGTCCCTGTTAAAAAGGGTGCCTGTTTTAGGGACTTCAAAGGTGTCTGGGTTCGACCTGATAACAAAGTTGAATGGGATCCTGATGTAGAACGCGTGTATCTGCCTTCAGGAAAGCCACTGGTACCTGATTATGCAATGACTTTTGTCGGAGGAACTTCTTCGAAACCATTTAGTCGGTTATGGTGGGATGAAACTGTACCTACTGTAGTTACCAGGGCTGAACCCCATAATCAGACTATCACACATCCTGAACAAAACAGGGTACTCACAATCCGTGAGAATGCCAGACTCAAGATCCATGA

**▶Clustal omega result of the forward and reverse complement sequences**

forward ------------------------------------------------------------ 0

reverse CACCCCCCCCCGACCCAGAAGCTATAACCCTCCTACACCTACCCCTTAGGCAAGAACATA 60

forward ------------------------------------------------------------ 0

reverse GGCCCACCCCCCCCCCCCATCCCCACCGCACCTCATCCGAAACCCCCCCCCCACTACCTT 120

forward ------------------------------------------------------------ 0

reverse CCCGCCACCCCGCCCACTCTCTACCCCCCCCCCCCCCCCCCTCATCTTCTCCCCCCCATT 180

forward -----------------------------------AGGGTGCTGTTTTAGGGATTCAAGG 25

reverse TTTCAACGTTTTTTTCCATGTCCCTGTTAAAAAGGGTGCCTGTTTTAGGGACTTCAAAGG 240

* * ** * * ****

forward TGTCTGGGTTCGACCTGATAACAAAGTTGAATGGGATCCTGATGTAGAACGCGTGTATCT 85

reverse TGTCTGGGTTCGACCTGATAACAAAGTTGAATGGGATCCTGATGTAGAACGCGTGTATCT 300

************************************************************

forward GCCTTCAGGAAAGCCACTGGTACCTGATTATGCAATGACTTTTGTCGGAGGAACTTCTTC 145

reverse GCCTTCAGGAAAGCCACTGGTACCTGATTATGCAATGACTTTTGTCGGAGGAACTTCTTC 360

************************************************************

forward GAAACCATTTAGTCGGTTATGGTGGGATGAAACTGTACCTACTGTAGTTACCAGGGCTGA 205

reverse GAAACCATTTAGTCGGTTATGGTGGGATGAAACTGTACCTACTGTAGTTACCAGGGCTGA 420

************************************************************

forward ACCCCATAATCAGACTATCACACATCCTGAACAAAACAGGGTACTCACAATCCGTGAGAA 265

reverse ACCCCATAATCAGACTATCACACATCCTGAACAAAACAGGGTACTCACAATCCGTGAGAA 480

************************************************************

forward TGCCAGACTTCAAGGATTTCCAGATTATTATCAGCTCGAAAGCCCCAATAAATAAGGTTT 325

reverse TGCCAGACTCAAGATCCATGA--------------------------------------- 501

********* * *

forward AGTTAAATTGTGTTGGTGTGGTGGGTCGTTGGTGGTGTGGTGGGTTGTGGGGGGGGTTGT 385

reverse ------------------------------------------------------------ 501

forward GTGAGGTTGTTGGTTGGGGGGGTTTTGGGGGGTGGTGTGGCAGGTTTTGGGGTGGGTTGG 445

reverse ------------------------------------------------------------ 501

forward GGTTTGTGGGGGGGGGGTGGGTGGTGGGGTGTTTTTGGG 484

reverse --------------------------------------- 501

**Amplified sequence regions of the contig ‘Contig2268’ (Primer for, rev, rev2)**

> Contig2268
AGGCCCGTGATGAAATGCCATATGGTAGGAAGCCCGAAACTGAGTTGCAAAGGTTCATGCGATTAAAGAAGGAAGAGATGCCAGGTTTTTCAGCACTGGGATCAGAACCATCAAAACCTACTCTTTTTGATCATCGTCCACTTCGACTGAACGATGATGATTATCAACGTGTCTGCCATGTCCCTGTTAAAAAGGGTGCCTGTTTTAGGGACTTCA**AAGGTGTCTGGGTTCGACCTGATAACAAAGTTGAATGGGATCCTGATGTAGAACGCGTGTATCTGCCTTCAGGAAAGCCACTGGTACCTGATTATGCAATGACTTTTGTCGGAGGAACTTCTTCGAAACCATTTAGTCGGTTATGGTGGGATGAAACTGTACCTACTGTAGTTACCAGGGCTGAACCCCATAATCAGACTATCACACATCCTGAACAAAACAGGGTACTCACAATCCGTGAGAATGCCAGACTTCAAGGATTTCCAGATTATTATCAGCT**TCGTGGCCCAATTAAAGAAAGGTACATTCAAGTAGGGAATGCTGTTGCCGTCCCGGTTGCAAGAGCCCTAGGTTTCTCTCTAGGGTTAGCTGCTAAAGGTTCATCTAGTGAGCAACCTTTGCTCACATTGCCCAGGAAGTTTCCCAACATTCAGGACCACCCATCTCCTGCCATTGATGAGGATTGAGGAACACATTAGTTATTGTTG

**▶Clustal omega result of the amplified sequence and the Forward primer sequence**

MET1 AGGCCCGTGATGAAATGCCATATGGTAGGAAGCCCGAAACTGAGTTGCAAAGGTTCATGC 60

forward ------------------------------------------------------------ 0

MET1 GATTAAAGAAGGAAGAGATGCCAGGTTTTTCAGCACTGGGATCAGAACCATCAAAACCTA 120

forward ------------------------------------------------------------ 0

MET1 CTCTTTTTGATCATCGTCCACTTCGACTGAACGATGATGATTATCAACGTGTCTGCCATG 180

forward ------------------------------------------------------------ 0

MET1 TCCCTGTTAAAAAGGGTGCCTGTTTTAGGGACTTCAAAGGTGTCTGGGTTCGACCTGATA 240

forward ---------------AGGGTGCTGTTTTAGGGATTCAAGGTGTCTGGGTTCGACCTGATA 45

* * ** * * ************************

MET1 ACAAAGTTGAATGGGATCCTGATGTAGAACGCGTGTATCTGCCTTCAGGAAAGCCACTGG 300

forward ACAAAGTTGAATGGGATCCTGATGTAGAACGCGTGTATCTGCCTTCAGGAAAGCCACTGG 105

************************************************************

MET1 TACCTGATTATGCAATGACTTTTGTCGGAGGAACTTCTTCGAAACCATTTAGTCGGTTAT 360

forward TACCTGATTATGCAATGACTTTTGTCGGAGGAACTTCTTCGAAACCATTTAGTCGGTTAT 165

************************************************************

MET1 GGTGGGATGAAACTGTACCTACTGTAGTTACCAGGGCTGAACCCCATAATCAGACTATCA 420

forward GGTGGGATGAAACTGTACCTACTGTAGTTACCAGGGCTGAACCCCATAATCAGACTATCA 225

************************************************************

MET1 CACATCCTGAACAAAACAGGGTACTCACAATCCGTGAGAATGCCAGACTTCAAGGATTTC 480

forward CACATCCTGAACAAAACAGGGTACTCACAATCCGTGAGAATGCCAGACTTCAAGGATTTC 285

************************************************************

MET1 CAGATTATTATCAGCTTCGTGGCCCAATTAAAGAAAGGTACATTCAAGTAGGGAATGCTG 540

forward CAGATTATTATCAGCTCGAAAGCCCCAATAAATAAGGTTTAGTTAAATTGTGTTGGTGTG 345

**************** **** * **** ** * * ** ** * * **

MET1 TTGCCGTCCCGGTTGCAAGAGCCCTAGGTTTCTCTCTAGGGTTAGCTGCTAAAGGTTCAT 600

forward GTGGGTCGTTGGTGGTGTGGTGGGTT-----GTGGGGGGGGTT----GTGTGAGGTTGTT 396

** *** * * * * ***** * ***** *

MET1 CTAGTGAGCAACCTTTGCTCACATTGCCCAGGAAGTTTCCCAACATTCAGGACCACCCAT 660

forward GGTTGGGGGGGTTTTGG-----------GGGGTGGTGTGGCAGGTTTTGGGGTGG----- 440

* * ** * ** ** * ** ** **

MET1 CTCCTGCCATTGATGAGGATTGAGGAACACATTAGTTATTGTTG-- 704

forward --GTTGGGGTTTGTGGGGGGGGGGTGGGTGGTGGGGTGTTTTTGGG 484

** ** ** ** * * * * * ** ***

The synthesized sequence has overlapped with the MET1 contig by 280 base pairs.

**▶BLASTX search result of Contig2268 and MET1 (AT5G49160)**

>AT5G49160.1 | Symbols: MET1, MET2, METI, DDM2, DMT01, DMT1 | methyltransferase 1

| chr5:19932501-19938186 FORWARD LENGTH=1534

Length = 1534

Score = 178 bits (451), Expect = 2e-44

Identities = 129/383 (33%), Positives = 178/383 (46%), Gaps = 8/383 (2%)

Frame = +3

Query: 1170 LPLPGDADVICGGPPCQGISGFNRFRNTDSPLDDPKNQQLVVYMDIVNYLKPRYVLMENV 1349

LPLPG D I GGPPCQG SG NRF S + + ++ ++ +Y +PRY L+ENV

Sbjct: 1183 LPLPGQVDFINGGPPCQGFSGMNRFNQ--SSWSKVQCEMILAFLSFADYFRPRYFLLENV 1240

Query: 1350 VDILRFAGGFLARYALGRLVGMNYQARMGLMAAGAYGLPQFRMRMFMWGAHNMEKLPQYP 1529

+ F G + L L+ M YQ R G++ AGAYG+ Q R R F+W A E LP++P

Sbjct: 1241 RTFVSFNKGQTFQLTLASLLEMGYQVRFGILEAGAYGVSQSRKRAFIWAAAPEEVLPEWP 1300

Query: 1530 LPTHNVVVRGNTPTEFE-SNAVAYDEGHGVELE---KKLFLGDAISDLPPVENNEARDEM 1697

P H V G + S + Y L + + + D I DLP VEN ++R

Sbjct: 1301 EPMH---VFGVPKLKISLSQGLHYAAVRSTALGAPFRPITVRDTIGDLPSVENGDSRTNK 1357

Query: 1698 PYGRKPETELQRFMRLKKEEMPGFSALGSEPSKPTLFDHRPLRLNDDDYQRVCHVPVKKG 1877

Y + Q+ +R + L DH +N+ + R +P + G

Sbjct: 1358 EYKEVAVSWFQKEIR---------------GNTIALTDHICKAMNELNLIRCKLIPTRPG 1402

Query: 1878 ACFRDFKGVWVRPDNKVEWDPDVERVYLPSGKPLVPDYAMTFVGGTSSKPFSRLWWDETV 2057

A + D P KV +P P + + G + RL W

Sbjct: 1403 ADWHDL------PKRKVTLSDGRVEEMIPFCLPNTAERHNGWKG-----LYGRLDWQGNF 1451

Query: 2058 PTVVTRAEPHNQT--ITHPEQNRVLTIRENARLQGFPDYYQLRGPIKERYIQVGNAVAVP 2231

PT VT +P + HPEQ+R+LT+RE AR QGFPD Y+ G I ++ Q+GNAV P

Sbjct: 1452 PTSVTDPQPMGKVGMCFHPEQHRILTVRECARSQGFPDSYEFAGNINHKHRQIGNAVPPP 1511

Query: 2232 VARALGFSL--GLAAKGSSSEQP 2294

+A ALG L L K S QP

Sbjct: 1512 LAFALGRKLKEALHLKKSPQHQP 1534

**Sequence 4**

Target gene = DDM1 gene


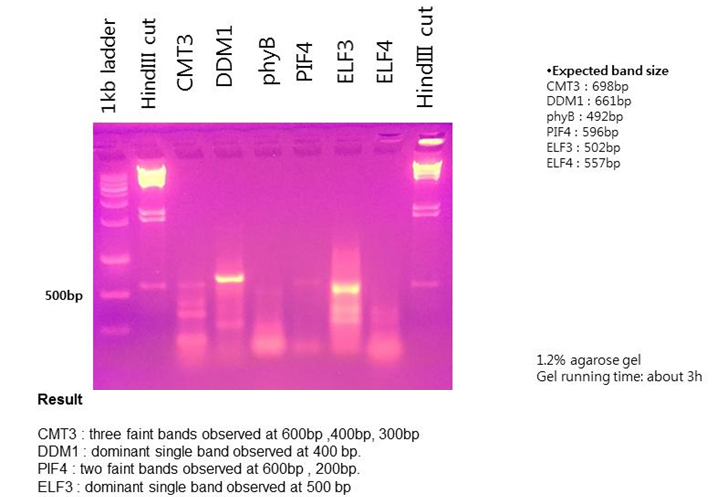


- **DDM1 Sanger Sequencing result**

**Forward result [206bp]**

TATTTCGTTTCTGCACTGAGTATACAATTGAGGAGAAAGTTATTGAGAGAGCTTATAAAAAACTTGCACTTGATGCTTTAGTTATCCAGCAAGGACGACTAGCTGAGCAAAAGACTGTTAACAAAGATGAGCTGTTACAAATGGTGAGGTTTGGTGCTGAAATGGTTTTCAGTTCCAAAGATAGCACAATCAAGACGAGGATAGAA

**Reverse result [193bp]**

CTTCTCACAGGAATGGGCAAAAGAAAGAAGTTCAAGTTTTCCGTTTCTGCACTGAGTATACAATTGAGGAGAAAGTTATTGAGAGAGCTTATAAAAAACTTGCACTTGATGCCTTAGTTATCCAGCAAGGACGACTAGCTGAGCAAAAGACTGTTAACAAAGATGAGCTGTTGCAAATGGTGAGGTTGGATAG

**▶Clustal omega result of the forward and reverse complement sequences**

forward ------------------------------------TATTTCGTTTCTGCACTGAGTATA 24

reverse CTTCTCACAGGAATGGGCAAAAGAAAGAAGTTCAAGTTTTCCGTTTCTGCACTGAGTATA 60

* ** *******************

forward CAATTGAGGAGAAAGTTATTGAGAGAGCTTATAAAAAACTTGCACTTGATGCTTTAGTTA 84

reverse CAATTGAGGAGAAAGTTATTGAGAGAGCTTATAAAAAACTTGCACTTGATGCCTTAGTTA 120

**************************************************** *******

forward TCCAGCAAGGACGACTAGCTGAGCAAAAGACTGTTAACAAAGATGAGCTGTTACAAATGG 144

reverse TCCAGCAAGGACGACTAGCTGAGCAAAAGACTGTTAACAAAGATGAGCTGTTGCAAATGG 180

**************************************************** *******

forward TGAGGTTTGGTGCTGAAATGGTTTTCAGTTCCAAAGATAGCACAATCAAGACGAGGATAG 204

reverse TGAGGTTGGATAG----------------------------------------------- 193

******* * *

forward AA 206

reverse -- 193

**Amplified sequence regions of the contig ‘TRINITY_DN215566_c4_g6_i1’ (Primer for, for2, rev)**

AAGCCAGGAAGTGAGAAATTTGTCTTCTTATTGTCAACTAGAGCTGGTGGCCTTGGAATTAATCTTGCGACTGCTGATATTGTTATTCTATATGACAGTGACTGGAACCCACAAGTTGATTTACAGGCTCAGGACCGTGCTCACAGGATTGGGCAAAAGAAAGAAGTTCAAGTTTTC**CGTTTCTGCACTGAGTATACAATTGAGGAGAAAGTTATTGAGAGAGCTTATAAAAAGCTTGCACTTGATGCTTTAGTTATCCAGCAAGGACGACTAGCTGAGCAAAAGACTGTTAACAAAGATGAGCTATTGCAAATGGTTAGGTTTGGTGCTGAAATGGTTTTCAGTTCCAAAGATAGCACAATCA**CAGACGAGGATATAGACAGGATCATTGCTAAAGGAGAAGAAGCAACAGCTGA

▶Clustal omega result of the amplified sequence and the Forward primer sequence

expected AAGCCAGGAAGTGAGAAATTTGTCTTCTTATTGTCAACTAGAGCTGGTGGCCTTGGAATT 60

forward ------------------------------------------------------------ 0

expected AATCTTGCGACTGCTGATATTGTTATTCTATATGACAGTGACTGGAACCCACAAGTTGAT 120

forward ------------------------------------------------------------ 0

expected TTACAGGCTCAGGACCGTGCTCACAGGATTGGGCAAAAGAAAGAAGTTCAAGTTTTCCGT 180

forward ----------------------------------------------------TATTTCGT 8

* ** ***

expected TTCTGCACTGAGTATACAATTGAGGAGAAAGTTATTGAGAGAGCTTATAAAAAGCTTGCA 240

forward TTCTGCACTGAGTATACAATTGAGGAGAAAGTTATTGAGAGAGCTTATAAAAAACTTGCA 68

***************************************************** ******

expected CTTGATGCTTTAGTTATCCAGCAAGGACGACTAGCTGAGCAAAAGACTGTTAACAAAGAT 300

forward CTTGATGCTTTAGTTATCCAGCAAGGACGACTAGCTGAGCAAAAGACTGTTAACAAAGAT 128

************************************************************

expected GAGCTATTGCAAATGGTTAGGTTTGGTGCTGAAATGGTTTTCAGTTCCAAAGATAGCACA 360

forward GAGCTGTTACAAATGGTGAGGTTTGGTGCTGAAATGGTTTTCAGTTCCAAAGATAGCACA 188

***** ** ******** ******************************************

expected ATCACAGACGAGGATATAGACAGGATCATTGCTAAAGGAGAAGAAGCAACAGCTGA 416

forward ATCAAGACGAGGATAGAA-------------------------------------- 206

**** * *

The synthesized sequence has overlapped with the DDM1 contig by 187 base pairs.

**▶BLASTX search result of TRINITY_DN215566_c4_g6_i1 and DDM1 (AT5G66750)**

>[AT5G66750](http://www.arabidopsis.org/servlets/TairObject?type=locus&name=AT5G66750).1 | Symbols: DDM1, CHR01, CHR1, CHA1, SOM4, SOM1, ATDDM1 | chromatin

remodeling 1 | chr5:26649050-26652869 FORWARD LENGTH=764

Length = 764

Score = 122 bits (306), Expect = 8e-29

Identities = 61/132 (46%), Positives = 89/132 (67%), Gaps = 5/132 (3%)

Frame = +1

Query: 22 VFLLSTRAGGLGINLATADIVILYDSDWNPQVDLQAQDRAHRIGQKKEVQVFRFCTEYTI 201

+FLLSTRAGGLGINL AD ILYDSDWNPQ+DLQA DR HRIGQ K V V+R T +I

Sbjct: 595 IFLLSTRAGGLGINLTAADTCILYDSDWNPQMDLQAMDRCHRIGQTKPVHVYRLSTAQSI 654

Query: 202 EEKVIERAYKKLALDALVIQQGRLAEQKT-----VNKDELLQMVRFGAEMVFSSKDSTIT 366

E +V++RAY KL L+ +VI QG+ +++ + ++++L +++ + I+

Sbjct: 655 ETRVLKRAYSKLKLEHVVIGQGQFHQERAKSSTPLEEEDILALLKEDETAEDKLIQTDIS 714

Query: 367 DEDIDRIIAKGE 402

D D+DR++ + +

Sbjct: 715 DADLDRLLDRSD 726

**Sequence 5**

Target gene = Chlorophyll a-b binding protein


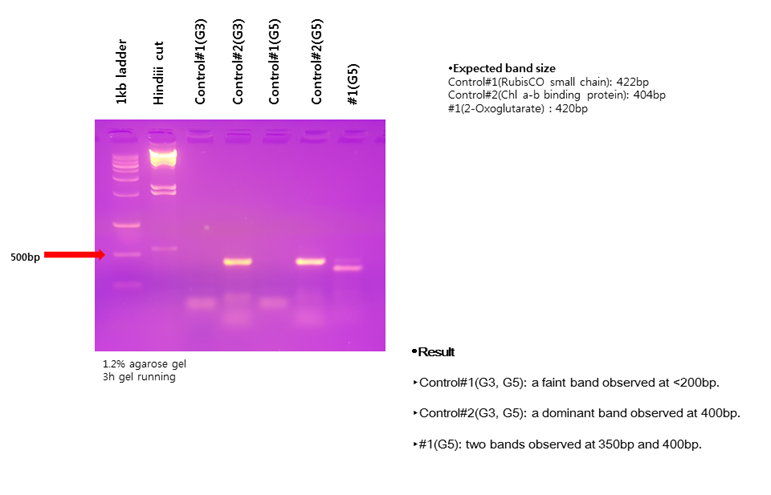


- **Chlorophyll a-b Sanger Sequencing result**

**Forward result [375 bp]**

>20170918_Chl_20170918_Chl47
CGTTACTAACTCAATGAGTATACTCCTCACTTTCTTCGGATCATTGGAGTTGCCTAATGCATCAGAAGCTCAACCGGGGTACTTTTTCTTCTCGGAATCTTTCTCCATGCTTCTTTGATGCTCAACCAATGCCATGAAGAGGAATTCGATTATGGGCTGGTGGGAAGGGGACCCCCCGGTACTGCCTTTTTGGTGATCGAAGTCTGTTCCCCGGCATTTGCTGAGCACTCTTGAGCCTGGAGAAAGATTCCGAGAAGAAAAAGTACCCGCGTGGAGCTTTTGACCCAGTAGGCTACTCCCAGGACCCAGTGGAAATTCGAGGACTCCATGTCTCGGAGGTGGTTTTTGGGCATCTTGCATTGCTGGAAATTCGTC

**Reverse result [365bp]**

**>**20170918_Chl_20170918_Chl48
TACGAGTTCGGCAATGCAAGATGCCCAAAAACCACCTCCGAGACATGGAGTCCTCGAATTCCACGGGTCCTGGGAGTAGCCTAATGGGTCAAAAGCTCCACCCGGGTACTTTTTCTTCTCGGAATCTTTCTCCAGGCTCAAGAGTGCTCAGCAAATGCAAGGGAGAGGAATTCGATCACCAAAATGGCAGTAACGGTACCTCACGTGACCGGCAGCCCATGTTGGAGTCCTCTTCCGGGGATTGCTTGAGATCAAAGACGCATGGAGAAAGATTCAAGAAGTAAATCAGGCCCCTGAACTCCTGGTACGAAAGGCAGCACCCCTAGTCAGACGAGAAGTCAGATACATGATCATCAGATAAGACG

**▶Clustal omega result of the forward and reverse complement sequences**

forward ---------------------------CGTTACTAACTCAATGAGTATACTCCTCACTTT 33

reverse TACGAGTTCGGCAATGCAAGATGCCCAAAAACCACCTCCGAGACATGGAGTCCTCGAATT 60

* * * * * ***** **

forward CTTCGGATCATTGGAGTTGCCTAATGCATCAGAAGCTCAACCGGGGTACTTTTTCTTCTC 93

reverse CCACGGGTCCTGGGAGTAGCCTAATGGGTCAAAAGCTCCACCCGGGTACTTTTTCTTCTC 120

* *** ** * ***** ******** *** ****** *** *****************

forward GGAATCTTTCTCCATGCTTCTTTGATGCTCAACCAATGCCATGAAGAGGAATTCGATTAT 153

reverse GGAATCTTTCTCCAGGCTCAAGAG-TGCTCAGCAAATGCAAGGGAGAGGAATTCGATCAC 179

************** *** * ****** * ***** * * ************* *

forward GGGCTGGTGGGAAGGGGACCCCCCGGTACTGCCTTTTTGGTGATCGAAGTCTGTTCCCCG 213

reverse CAAAATGGCAGTAACGGTACCTCACGTGACCGGCAGCCCATGTT-GGAGTCCTCTTCCGG 238

* * * ** ** * ** ** * * **** * ** *

forward GCATTTGCTGAGCACTCTTGAGCCTGGAGAAAGATTCCGAGAAGAAAAAGTACCCGCGTG 273

reverse GGATTGCTTGAGATCAAAGACGCATGGAGAAAGATTCAAGAAGTAAATCAGGCCCCTGAA 298

* *** **** * ** ************* * *** *** *

forward GAGCTTTTGACCCAGTAGGCTACTCCCAGG-ACCCAGTGGAAATTCGAGGACTCCATGTC 332

reverse CTCCTG---GTACGAAAGGCAGCACCCCTAGTCAGACGAGAAGTCAGATACATGATCATC 355

** * **** * *** * * *** * ** * **

forward TCGGAGGTGGTTTTTGGGCATCTTGCATTGCTGGAAATTCGTC 375

reverse AGATAAGACG--------------------------------- 365

**Ginseng pseudo gene sequence**

>TRINITY_DN169002_c1_g1_i1 (for primer/ rev primer) [amplification size 404bp]
TAAATATTGAACTTTTAAGTATGATCAGGAGGAATTAGTGCTGGTTTATTTTCAATTCGTAGACGATATTTATTTGAAGCATTCTTCGATCATCACCACTTAAGTAGCATCATCGCGAACAATTAAGTTTATGAACAGCAGATAAAATTGGAACAATACGCAGAACAGGATGTATTAGCAAAAGTTCCTGGTAAGTAAAATTACTCTTCAAACTTAAAAAAGGACAATCATATACAGATACACAAGTTCACTTCATTAGGTAGCATAAATAATGTCACATGAGTTTTACAATGAGGCTATTTCATATTAAACTGGACTCCATCAAGGCAAATTAAAGTCCTCTGGGGATAATGACATTTCCGATGTTGTTGTGCCATGGGTCAGCCAAGTGAGTTGCCAAGTTCTCCAATGGTCCTGTTCCTGGGTATGCAGACTGTTGAACACAGAATCCCACGAATGCCAGCAATGCAAGACGCCCATTTTTGACCTCCTTGACCTTATACTCCTCGAA**TTTCTTCGGGTCCTTGGAGTAGCCTAATGGGTCAAAAGCTCCACCGGGGTACTTTTTCTTCTCGGAATCTTTCTCCATGCTTCTTTGGTGCTCAACAAATGCAATGGAGAGGAATTCGATTACCAAAATGGTGGGAAGGGTACCCCATGGGACTGGGTTGCCCAAGTAGGTTGCTTGTCCTCCGGGGATTGCTGCCCACTCTTGAGCCTGTACCCAGTTGCCCAAACCCAAAGCCTCAGGC**ACCAAAATCCCTGGAACAGCAAGCATGGCCCATCTGCAGTGGATGAGTTCAGACTCCTTGAATCTCTCAAGGTTTTCCGGGACTGAACCAAGTCCTAGCGGGTCGAACCCGAAATCTCCGGGTGCTGATCCGTCAAGGTAGGGTGGCCGGGGCTGGCCAGGCATCCAATCGGAAGTCATGGTGTAGCGGGATGTTGTGCCATTGGCACTAACCAAAGACTGCGGCAATGCCGCCGCAAATTTAGACTTTGAAGAGGAAAGAACCGAAGGGAAGACGGCGGCGATGCCACAGCTCATCAGTGCATTTGTGGCCATTCTCAGAAATATTGTAATCGTCTAGCTGAGAATACTAGACTAAAATGAAGAAAAAAGAGAGAGAGAAAGAAGGCTGTGAGGGATGAAGAAGTGAGTGGCAGGGGACATGTACAAGTATATGGAAGAAGGGGGATATAGGCAAATCCAGATATTGATTGTAATTGGTTAAGGGTAGTTTTCTTTGGTTTTGATTGGTCCACGTGGCACCTGTCAATCCATATGGATACCTATCTGGAATATCTAATGGATGTGGATCTTAAATTTGTGTAGATTTTGGATTGTTTTCTATGTAGGAAAATTAGGGAGGAGGGAGGAGGGAGGAGGGAGGAGGGAGGAG

**▶Clustal omega result of the amplified sequence and the Forward primer sequence**

amplified ACGAATGCCAGCAATGCAAGACGCCCATTTTTGACCTCCTTGACCTTATACTCCTCGAAT 60

forward -----------------------------CGTTACTAACTCAATGAGTATACTCCTCACT 31

* ** ** * * * *

amplified TTCTTCGGGTCCTTGGAGTAGCCTAATGGGTCAAAAGCTCCACCGGGGTACTTTTTCTTC 120

forward TTCTTCGGATCATTGGAGTTGCCTAATGCATCAGAAGCTCAACCGGGGTACTTTTTCTTC 91

******** ** ******* ******** *** ****** *******************

amplified TCGGAATCTTTCTCCATGCTTCTTTGGTGCTCAACAAATGCAATGGAGAGGAATTCGATT 180

forward TCGGAATCTTTCTCCATGCTTCTTTGATGCTCAACCAATGCCATGAAGAGGAATTCGATT 151

************************** ******** ***** *** **************

amplified ACCAAAATGGTGGGAAGGGTACCCCATGGGACTGGGTTGCCCAAGTAGGTTGCTTGTCCT 240

forward ATGGGCTGGTGGGAAGG-GGACCCCCCGGTACTGCCTTTTTGGTGATCGAAGTCTGTTCC 210

* * ** * * * ***** ** **** ** * * * *** *

amplified CCGGGGATTGCTGCCCACTCTTGAGCCTGTACCCAGTTGCCCAAACCCAAAGCCTCAGGC 300

forward CCGGCATTTGCTGAGCACTCTTGAGCCTGGAGAAAGATTCCGAGAAGAAAAAGTACCCGC 270

**** ****** ************** * ** * ** * * *** * **

amplified ACCAAAATCCCTGGAACAGCAAGCATGGCCCATCTGCAGTGGATGAGTTCAGACTCCTTG 360

forward GTGGAGCTTTTGACCCAGTAGGCTACTCCCAGGACCCAGTGGAAATTCGAGGACTCCATG 330

* * * ** ******* ****** **

amplified AATCTCTCAAGGTTTTCCGGGACTGAACCAAGTCCTAGCGGGTC--- 404

forward TCTCGGAGGTGGTTTTTGGGCATCT--TGCATTGCTGGAAATTCGTC 375

** ****** ** * * * ** * **

The synthesized sequence has overlapped with the Chlorophyll a-b contig by 241 base pairs.

**▶BLASTX search result of TRINITY_DN169002_c1_g1_i1 and Chlorophyl a-b binding protein (AT1G29920)**

>[AT1G29920](http://www.arabidopsis.org/servlets/TairObject?type=locus&name=AT1G29920).1 | Symbols: CAB2, AB165, LHCB1.1 | chlorophyll A/B-binding protein 2

| chr1:10475089-10475892 REVERSE LENGTH=267

Length = 267

Score = 120 bits (301), Expect = 2e-27

Identities = 78/191 (40%), Positives = 99/191 (51%), Gaps = 13/191 (6%)

Frame = -2

Query: 898 PPYLDGSAPGDFGFDPLGLGSVPENLERFKESELIHCRWAMLAVPGILVPEAL------- 740

P YL G PGD+G+D GL + PE R +E E+IH RWAML G + PE L

Sbjct: 67 PSYLTGEFPGDYGWDTAGLSADPETFARNRELEVIHSRWAMLGALGCVFPELLARNGVKF 126

Query: 739 GLGNWVQAQEWAAIPGGQATYLGNP--VPWGTLPTILVIEFLSIAFVEHQRSMEK----D 578

G W +A GG YLGNP V ++ I + + + VE R +

Sbjct: 127 GEAVWFKAGSQIFSDGG-LDYLGNPSLVHAQSILAIWATQVILMGAVEGYRVAGNGPLGE 185

Query: 577 SEKKKYPGGAFDPLGYSKDPXXXXXXXXXXXKNGRLALLAFVGFCVQQSAYPGTGPLENL 398

+E YPGG+FDPLG + DP KNGRLA+ + GF V Q+ G GP+ENL

Sbjct: 186 AEDLLYPGGSFDPLGLATDPEAFAELKVKELKNGRLAMFSMFGFFV-QAIVTGKGPIENL 244

Query: 397 ATHLADPWHNN 365

A HLADP +NN

Sbjct: 245 ADHLADPVNNN 255
